# Ceritinib induces ferroptosis via TRIM21-mediated GLUT1 ubiquitination and AMPK-driven metabolic reprogramming in breast cancer

**DOI:** 10.1016/j.isci.2026.115846

**Published:** 2026-04-22

**Authors:** Yao Chen, Changchang Liu, Ruonan Zhen, Jiarui Li, Yanran Li, Ziying Zhang, Jingying Zhang, Feng Gao, Yongli Bao, Luguo Sun, Shuyue Wang, Lihua Zheng, Ying Sun, Guannan Wang, Jiawei Li, Xiaoguang Yang, Zhenbo Song

**Affiliations:** 1 NMPA Key Laboratory for Quality Control of Cell and Gene Therapy Medicine Products, Northeast Normal University, Changchun 130024, China; 2 National Engineering Laboratory for Druggable Gene and Protein Screening, Northeast Normal University, Changchun 130117, China; 3 Hebei International Travel Healthcare Center (Shijiazhuang Customs Port Clinic), No. 35, Yangguang Road, Zhengding New District, Shijiazhuang, Hebei Province 050000, China; 4 Department of Railway Power Supply, Heilongjiang Communications Polytechnic, Harbin 150025, China

**Keywords:** Pharmacology, Molecular biology, Cancer

## Abstract

Although ceritinib, a potent ALK inhibitor, has been clinically approved for the treatment of non-small cell lung cancer, its potent cytotoxic activity against breast cancer cells and the underlying mechanisms remain largely unknown. We show that ceritinib inhibits proliferation and induces ferroptosis in breast cells. Mechanistically, ceritinib downregulates GLUT1, impairing glycolysis, inducing energy stress, and activating AMPK signaling. Activated AMPK promotes ferritinophagy, leading to iron accumulation, lipid peroxidation, and redox imbalance, ultimately triggering ferroptosis. Rescue experiments demonstrate that GLUT1 overexpression or AMPK inhibition attenuates these effects, confirming the role of the GLUT1-AMPK axis. At the molecular level, ceritinib promotes TRIM21-mediated ubiquitination and proteasomal degradation of GLUT1 through direct binding. *In vivo*, ceritinib suppresses tumor growth, while GLUT1 knockdown reduces its efficacy and ferroptotic response. These findings reveal a novel mechanism whereby ceritinib induces ferroptosis via GLUT1 downregulation and AMPK-dependent ferritinophagy, supporting its potential repurposing for breast cancer therapy.

## Highlights

• Ceritinib induces ferroptosis in breast cancer cells independently of ALK
• GLUT1 inhibition by ceritinib impairs glycolysis and activates AMPK signaling
• AMPK activation drives ferritinophagy and iron-dependent lipid peroxidation
• TRIM21-mediated GLUT1 degradation underlies ceritinib’s antitumor activity

## Introduction

Breast cancer continues to be the most commonly diagnosed malignancy and the leading cause of cancer-related deaths among women worldwide.^1^ Despite advances in multimodal therapies such as surgery, chemotherapy, radiotherapy, and molecularly targeted agents, significant clinical challenges remain, particularly drug resistance, treatment toxicity, and variable patient responses.^2^ These limitations underscore the urgent need to identify novel therapeutic targets and develop more effective treatment strategies. Drug repurposing of existing targeted agents represents a promising approach to address these unmet clinical needs.

Metabolic reprogramming is a fundamental hallmark of cancer that fuels tumor growth and progression through altered nutrient utilization. In breast cancer, this metabolic rewiring not only meets the bioenergetic and biosynthetic demands of rapidly proliferating cells but also creates unique therapeutic vulnerabilities. Importantly, targeting these metabolic alterations often leads to ferroptosis induction—a particularly effective strategy given that many metabolic inhibitors simultaneously disrupt redox homeostasis and iron metabolism. Ferroptosis is distinct from other classical forms of cell death, such as apoptosis, necrosis, and autophagy, in terms of its morphological, biochemical, immunological, and genetic features.^3^^,^^4^ Accumulating evidence demonstrates the tumor-suppressive potential of ferroptosis through multiple mechanisms, such as inhibiting proliferation,^3^^,^^5^^,^^6^ suppressing metastasis,^7^^,^^8^^,^^9^ reversing chemoresistance,^10^^,^^11^^,^^12^ and countering immune evasion,^13^^,^^14^ positioning it as a promising therapeutic approach.^3^^,^^15^ Central to ferroptosis regulation is ferritinophagy, an autophagic process mediated by nuclear receptor coactivator 4 (NCOA4) that degrades ferritin to release redox-active iron.^16^ This iron overload catalyzes Fenton reactions with hydrogen peroxide (H_2_O_2_), generating cytotoxic reactive oxygen species (ROS) and driving lipid peroxidation, for ferroptosis.^17^^,^^18^^,^^19^ This metabolic-ferroptosis axis suggests that targeted disruption of cancer metabolism could serve as a powerful strategy to induce ferroptosis while overcoming conventional therapeutic resistance. Notably, autophagy-mediated degradation of ferritin heavy chain 1 (FTH1) further amplifies the cascade.

Ceritinib, a second-generation anaplastic lymphoma kinase (ALK) inhibitor approved for ALK-rearranged non-small cell lung cancer (NSCLC), exhibits expanding therapeutic potential.^20^^,^^21^^,^^22^^,^^23^^,^^24^ However, emerging evidence suggests that ceritinib may exert antitumor activity in a broader range of malignancies, including those lacking ALK rearrangements or expression,^25^ suggesting off-target pathway engagement.^26^ Importantly, ceritinib has recently been identified as a modulator of CD39-mediated ATP degradation, suggesting its broader metabolic regulatory effects.^27^ While metabolic reprogramming is a well-established cancer hallmark, and several agents can simultaneously target metabolism and induce ferroptosis,^28^ whether ceritinib exerts its antitumor effects through metabolic modulation and ferroptosis induction in breast cancer remains unclear. Additionally, the underlying regulatory mechanisms of ceritinib in this context require further exploration.

This study elucidates a novel ALK-independent mechanism underlying ceritinib’s antitumor activity. Ceritinib induces TRIM21-mediated ubiquitination for GLUT1 degradation, disrupting glucose metabolism while triggering ferroptosis via iron dysregulation and redox imbalance. Our findings establish the Ceritinib/TRIM21/GLUT1 axis as a novel therapeutic pathway, which links metabolic targeting with ferroptosis, providing mechanistic insights for repurposing this clinically approved agent in breast cancer treatment.

## Results

### Ceritinib exerts cytotoxic effects and suppresses proliferation in breast cancer cells

**Figure 1:**
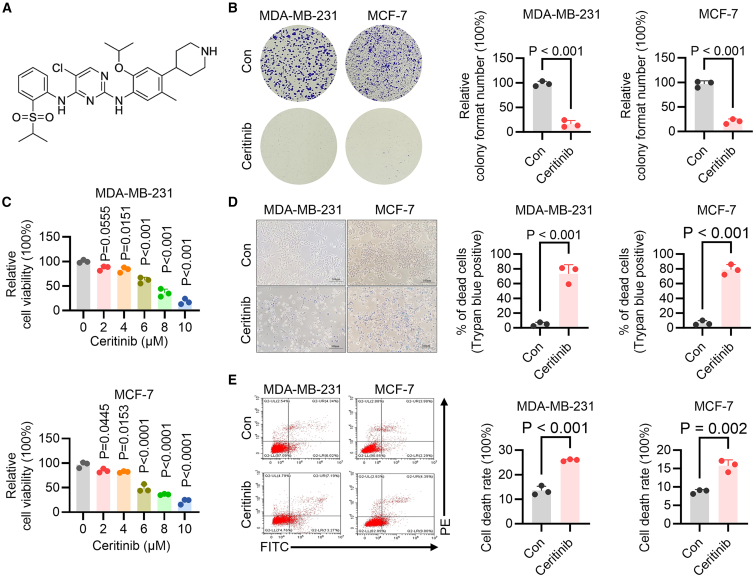
Ceritinib inhibits proliferation and induces death in breast cancer cells (A) Chemical structure of ceritinib. (B) Cell viability was assessed by MTT assay after 48 h of ceritinib treatment at various concentrations in MDA-MB-231 (top) and MCF-7 cells (bottom) (*n* = 3). (C) Representative images (left) and quantification of colony formation assays (right) in MDA-MB-231 and MCF-7 cells treated with vehicle (control) or ceritinib (*n* = 3). (D) Representative trypan staining images (left) and quantification of the ratio of dead cells (right) following ceritinib treatment (*n* = 3). (E) Flow cytometric analysis of cell death by annexin V-FITC/PI staining (left) and quantification of the ratio of dead cells (right) after ceritinib treatment in MDA-MB-231and MCF-7 cells (*n* = 3). Significance was assessed by Student’s *t* test, one-way ANOVA and Tukey’s post hoc test. The data are shown as the mean ± SEM.

Ceritinib, a clinically approved ALK inhibitor for NSCLC, has not been extensively investigated in the context of breast cancer. To elucidate its potential antitumor activity, we examined the effects of ceritinib on the viability and survival of MDA-MB-231 and MCF-7 breast cancer cells. Dose-dependent treatment with ceritinib resulted in a marked reduction of cell viability, as assessed by MTT assays, with both cell lines exhibiting pronounced sensitivity at concentrations ≥2 μM (Figures 1A and 1B). Consistent with this observation, colony formation assays revealed a significant impairment in long-term proliferative capacity following ceritinib exposure (Figure 1C). To determine whether the observed growth inhibition was attributable to cell death, we employed trypan blue exclusion and flow cytometric analysis of annexin V-fluorescein Isothiocyanate (FITC)/propidium iodide (PI) staining. Ceritinib treatment substantially increased the proportion of trypan blue-positive cells, indicating enhanced cytotoxicity (Figure 1D). Flow cytometry further confirmed a significant elevation in apoptotic and dead cell populations in both MDA-MB-231 and MCF-7 cells after treatment (Figure 1E). Collectively, these findings demonstrate that ceritinib exerts potent antitumor effects in breast cancer cells by suppressing proliferation and inducing apoptosis, highlighting its potential as a therapeutic agent beyond ALK-rearranged malignancies.

### Ceritinib induces ferroptosis through autophagy-dependent ferritin degradation

**Figure 2:**
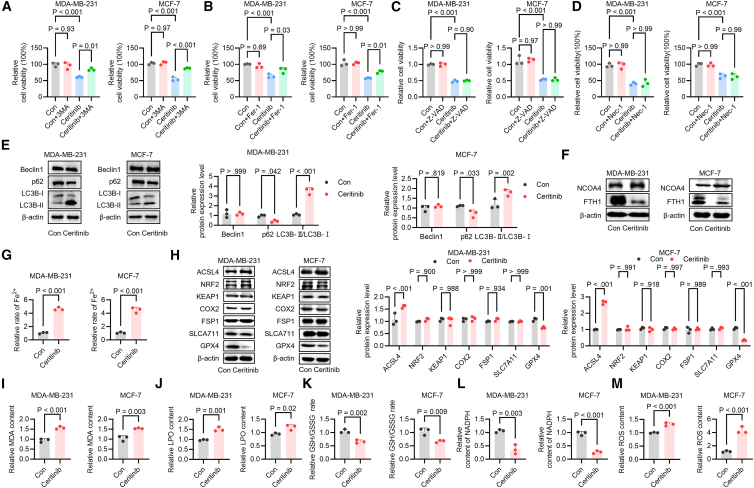
Ceritinib induces ferroptosis in breast cancer cells (A–D) Cell viability assays were conducted in MDA-MB-231 and MCF-7 cells treated with ceritinib alone or in combination with the ferroptosis inhibitor ferrostatin-1 (Fer-1, 15 μM), the autophagy inhibitor 3-methyladenine (3-MA, 25 μM), the apoptosis inhibitor Z-VAD-FMK (Z-VAD-FMK, 20 μM), or the necroptosis inhibitor Necrostatin-1 (Nec-1, 25 μM) (*n* = 3). (E) Western blot analysis was performed to evaluate autophagy-related proteins LC3, p62, and beclin1 in response to ceritinib treatment (*n* = 3). (F) Western blot was used to detect the expression of NCOA4 and FTH1 after ceritinib exposure (*n* = 3). (G) Intracellular Fe^2+^ concentrations were measured using a commercial iron assay kit (*n* = 3). (H) Western blot analysis was carried out to assess the expression levels of GPX4, SLC7A11, FSP1, ACSL4, NRF2, KEAP1, and COX2 (*n* = 3). (I and J) Lipid peroxidation levels were evaluated by measuring LPO and MDA contents in ceritinib-treated cells (*n* = 3). (K) The GSH/GSSG ratio was determined to assess the cellular redox status (*n* = 3). (L) NADPH levels were measured to evaluate the oxidative state of the cells treated with ceritinib (*n* = 3). (M) ROS accumulation was quantified using fluorescent probes in ceritinib-treated cells (*n* = 3). Significance was assessed by Student’s *t* test, one-way or two-way ANOVA and Tukey’s post hoc test. The data are shown as the mean ± SEM.

To elucidate the primary mechanism underlying ceritinib-induced cell death, MDA-MB-231 and MCF-7 cells were treated with ceritinib alone or in combination with pathway specific inhibitors: Ferrostatin-1 (Fer-1, ferroptosis inhibitor), 3-methyladenine (3-MA, autophagy inhibitor), Necrostatin-1 (Nec-1, necroptosis inhibitor) or Z-VAD-FMK (apoptosis inhibitor). Our data suggested that ceritinib markedly reduced cell viability in both lines, an effect that was substantially rescued by Fer-1 or 3-MA but remained insensitive to Nec-1 and Z-VAD-FMK treatment (Figures 2A–2D), indicating that ferroptosis and autophagy, rather than apoptosis and necroptosis are the dominant modes of cell death. Given the known crosstalk between autophagy and ferroptosis, we hypothesized that ceritinib-induced autophagy may facilitate ferroptotic cell death. Investigation of autophagic flux revealed that ceritinib increased LC3-II levels while reducing p62 expression, indicative of enhanced autophagosome formation (Figures 2E and S1A). Concurrently, the ferritinophagy receptor NCOA4 was upregulated, accompanied by decreased FTH1 levels, suggesting selective degradation of ferritin (Figures 2F and S1C). Consistent with this, intracellular ferrous iron (Fe^2+^) accumulation was observed following ceritinib treatment (Figure 2G), supporting the activation of ferritinophagy. Mechanistically, ceritinib also modulated key ferroptosis regulators: the ferroptosis-promoting enzyme Acyl-CoA synthetase long-chain family member 4 (ACSL4) was upregulated, whereas the lipid peroxide scavenger GPX4 was markedly downregulated, with other related proteins remaining largely unchanged (Figure 2H). Given the critical role of GPX4 as a key inhibitor of ferroptosis, we further investigated whether ceritinib affects GPX4 enzymatic activity in addition to its expression level. Our results revealed that ceritinib treatment significantly reduced GPX4 enzymatic activity in both MDA-MB-231 and MCF-7 cells (Figure S2). Functionally, ceritinib triggered hallmark ferroptotic events, including increased lipid peroxidation, as evidenced by elevated MDA and LPO levels (Figures 2I and 2J), reduced glutathione (GSH)/oxidized glutathione (GSSG) ratio (Figure 2K) and NADPH level (Figure 2L), and augmented ROS accumulation (Figure 2M). Together, these results indicate that ceritinib disrupts redox homeostasis through autophagy-mediated ferritin degradation, promoting iron-dependent lipid peroxidation and driving ferroptotic cell death in breast cancer cells.

### Ceritinib selectively impairs glycolysis through GLUT1 down-regulation

**Figure 3:**
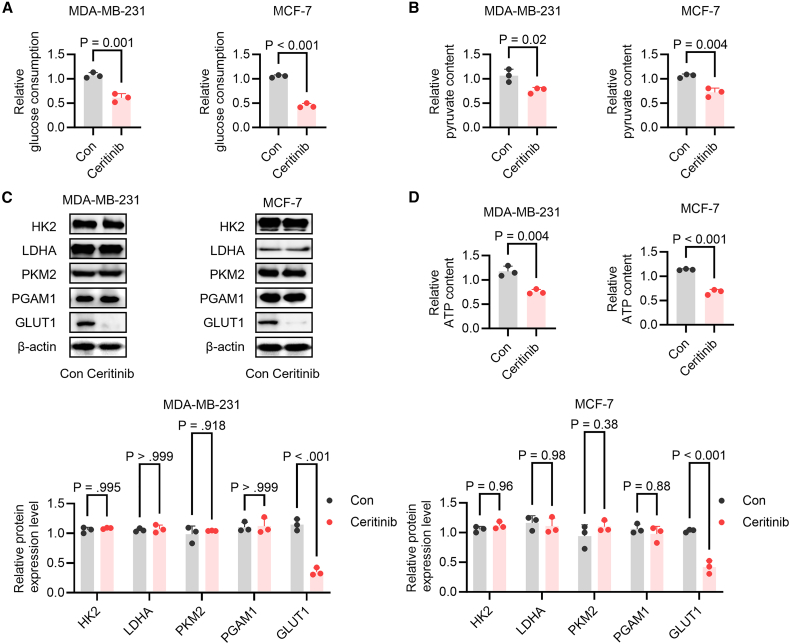
Ceritinib downregulates GLUT1 protein level and inhibits glycolysis in breast cancer cells (A) Glucose consumption was measured using a glucose assay kit in MDA-MB-231 and MCF-7 cells treated with ceritinib (*n* = 3). (B) Pyruvate content was determined with a colorimetric pyruvate detection assay under the same treatment conditions as described in (A) (*n* = 3). (C) Western blot was performed to assess the expression of HK2, LDHA, PKM2, PGAM1, and GLUT1 (*n* = 3). (D) Cellular ATP content was quantified using an ATP assay kit following ceritinib treatment (*n* = 3). Significance was assessed by Student’s *t* test, two-way ANOVA and Tukey’s post hoc test. The data are shown as the mean ± SEM.

Given the critical role of glycolytic metabolism in sustaining tumor growth and the known interplay between ferroptosis and cellular metabolism, we next investigated whether ceritinib-induced ferroptosis is associated with metabolic reprogramming. Treatment with ceritinib markedly reduced glucose uptake (Figure 3A) and intracellular pyruvate levels (Figure 3B) in MDA-MB-231 and MCF-7 cells, indicating a significant suppression of glycolytic flux. Notably, western blot analysis revealed that the expression of key glycolytic enzymes-including hexokinase 2 (HK2), lactate dehydrogenase A (LDHA), pyruvate kinase M2 (PKM2), and phosphoglycerate mutase 1 (PGAM1)-remained largely unchanged, whereas glucose transporter 1 (GLUT1) protein levels were substantially decreased (Figure 3C). These results suggest that ceritinib impairs glycolysis primarily through inhibition of GLUT1-mediated glucose uptake rather than direct modulation of enzymatic activity. Consistently, intracellular ATP content was significantly diminished following treatment (Figure 3D), reflecting pronounced energetic stress.

To determine whether ceritinib exerts broader metabolic effects, we assessed mitochondrial function. Protein levels of mitochondrial enzymes involved in the tricarboxylic acid cycle, including oxoglutarate dehydrogenase, pyruvate dehydrogenase (PDH), and isocitrate dehydrogenase 2 (IDH2), remained unchanged (Figure S3A). Similarly, activities of mitochondrial respiratory complexes I, III, and V (Figures S3B–D), as well as mitochondrial DNA (mtDNA) copy number (Figure S3E), were not significantly affected. Collectively, these findings indicate that ceritinib selectively disrupts glucose metabolism in breast cancer cells by downregulating GLUT1, while sparing mitochondrial oxidative function.

### Restoration of GLUT1 protects against ceritinib-induced ferroptosis

**Figure 4:**
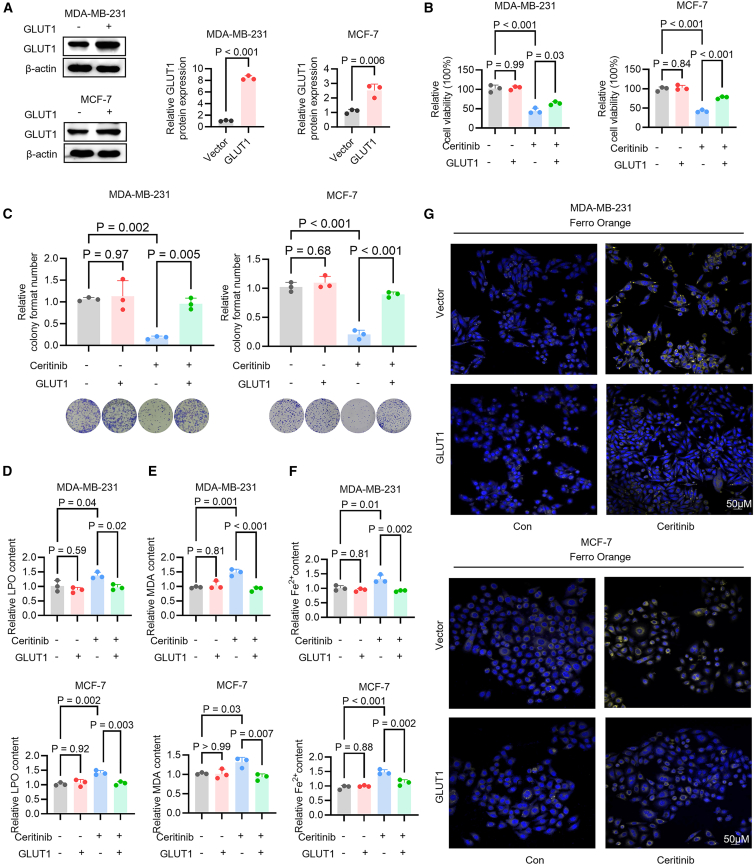
GLUT1 overexpression suppresses ceritinib-induced ferroptosis in breast cancer cells (A) Western blot analysis of GLUT1 protein levels in MDA-MB-231 and MCF-7 cells transfected with vector or GLUT1 plasmid to assess the transfection efficiency and GLUT1 expression (*n* = 3). (B) Cell viability analysis using MTT assay in MDA-MB-231 and MCF-7 cells under the indicated conditions, with ceritinib and/or GLUT1 overexpression (*n* = 3). (C) Representative images and quantification of colony formation in MDA-MB-231 and MCF-7 cells with or without ceritinib treatment, with or without GLUT1 expression (*n* = 3). (D) Quantification of LPO levels in MDA-MB-231 and MCF-7 cells treated with ceritinib in the presence or absence of GLUT1 overexpression (*n* = 3). (E) Measurement of MDA levels in MDA-MB-231 and MCF-7 cells under the indicated treatment conditions (*n* = 3). (F) Intracellular Fe^2+^ levels were measured in MDA-MB-231 and MCF-7 cells treated with ceritinib and/or GLUT1 overexpression (*n* = 3). (G) FerroOrange fluorescent probe was used to image Fe^2+^ accumulation in MDA-MB-231 and MCF-7 cells treated with ceritinib, with or without GLUT1 overexpression. Blue, DAPI-stained nuclei; yellow, FerroOrange-stained Fe^2+^. Scale bars, 50 μM. (*n* = 3). Significance was assessed by Student’s *t* test, one-way ANOVA and Tukey’s post hoc test. The data are shown as the mean ± SEM.

To elucidate the functional contribution of GLUT1 to ceritinib-induced ferroptosis, we generated GLUT1-overexpressing MDA-MB-231 and MCF-7 cell lines. Western blot analysis confirmed robust upregulation of GLUT1 in these engineered cells (Figure 4A). Functional assays revealed that GLUT1 overexpression markedly rescued the ceritinib-induced decline in cell viability, as assessed by MTT assays (Figure 4B), and substantially restored colony-forming ability (Figure 4C), highlighting the critical role of GLUT1 in mediating resistance to ceritinib cytotoxicity. Mechanistically, GLUT1 overexpression attenuated ceritinib-induced ferroptotic responses. Specifically, levels of LPO and MDA, key indicators of ferroptotic membrane damage, were significantly reduced compared to control cells (Figures 4D and 4E). Moreover, the accumulation of Fe^2+^, was substantially diminished in GLUT1-overexpressing cells (Figure 4F), an observation further corroborated by FerroOrange fluorescence imaging (Figures 4G and S4).

Together, these results demonstrate that GLUT1 downregulation is not merely a consequence of ceritinib treatment but is functionally indispensable for the induction of ferroptosis in breast cancer cells. By limiting glucose uptake and thereby perturbing cellular metabolism, loss of GLUT1 potentiates iron-dependent lipid peroxidation, linking metabolic suppression to ferroptotic cell death and establishing GLUT1 as a critical mediator of ceritinib sensitivity.

### Ceritinib triggers ferroptosis through GLUT1-mediated AMPK activation and ferritinophagy

Previous studies have demonstrated that AMP-activated protein kinase (AMPK) activation promotes intracellular Fe^2+^ accumulation by inducing ferritinophagy,^29^ while GLUT1 downregulation is known to trigger AMPK signaling.^30^ Based on these observations, we hypothesized that ceritinib-induced GLUT1 inhibition activates AMPK, which in turn drives ferritinophagy-mediated iron dysregulation, culminating in ferroptotic cell death.

**Figure 5:**
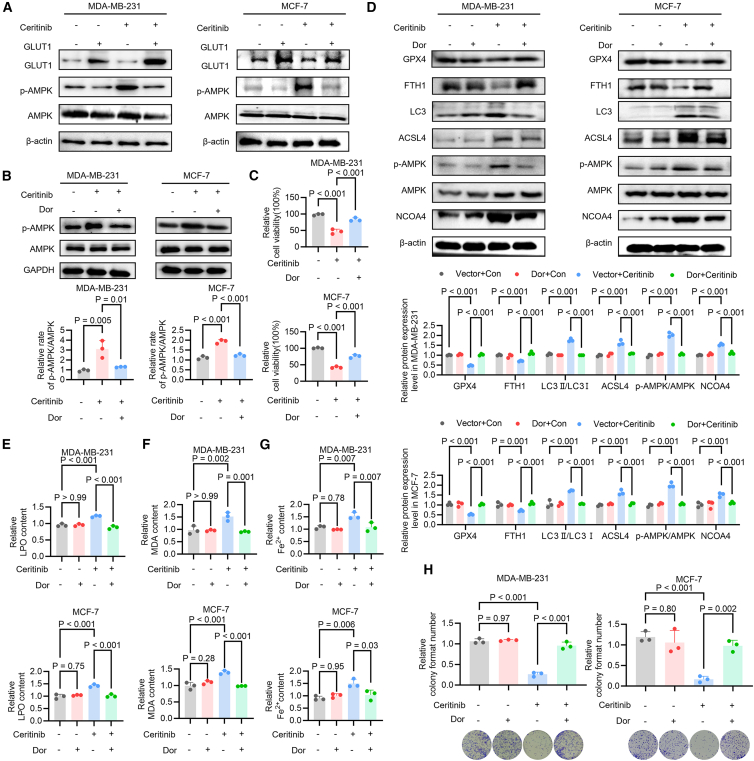
Ceritinib induces ferroptosis in breast cancer cells via GLUT1 downregulation and AMPK activation (A) Western blot analysis of GLUT1, *p*-AMPK, and AMPK levels in MDA-MB-231 and MCF-7 cells treated with ceritinib or control (*n* = 3). (B) Western blot analysis of *p*-AMPK level and quantification of the *p*-AMPK/AMPK ratio in MDA-MB-231 and MCF-7 cells treated with ceritinib or control, with or without AMPK inhibitor dorsomorphin (Dor) (*n* = 3). (C) Cell viability analysis using MTT assay in MDA-MB-231 and MCF-7 cells treated with ceritinib or control, with or without Dor (*n* = 3). (D) Western blot analysis of GPX4, FTH1, LC3B, and ACSL4 levels in MDA-MB-231 and MCF-7 cells treated with ceritinib and/or Dor (*n* = 3). (E) LPO levels in MDA-MB-231 and MCF-7 cells treated with ceritinib or control, with or without Dor (*n* = 3). (F) MDA levels in MDA-MB-231 and MCF-7 cells treated with ceritinib or control, with or without Dor (*n* = 3). (G) Intracellular Fe^2+^ levels in MDA-MB-231 and MCF-7 cells treated with ceritinib or control, with or without Dor (*n* = 3). (H) Colony formation assays in MDA-MB-231 and MCF-7 cells treated with ceritinib or control, with or without Dor (*n* = 3). Significance was assessed by 1-way ANOVA or two-way ANOVA and Tukey’s post hoc test. The data are shown as the mean ± SEM.

To test this, we first examined the relationship between GLUT1 expression and AMPK activation in ceritinib-treated MDA-MB-231 and MCF-7 cells. Ceritinib treatment markedly reduced GLUT1 protein levels while concurrently increasing AMPK phosphorylation (*p*-AMPK) (Figures 5A and S5). Notably, GLUT1 overexpression partially attenuated ceritinib-induced AMPK activation, suggesting that GLUT1 downregulation is upstream of AMPK signaling. Time-course analyses revealed that ceritinib-induced GLUT1 downregulation occurred early (4–6 h), preceding AMPK phosphorylation (8 h). NCOA4 upregulation and intracellular Fe^2+^ accumulation were observed at later stages (12–24 h). These observations suggest a sequential cascade whereby GLUT1 loss leads to AMPK activation, ultimately driving ferritinophagy-mediated ferroptosis (Figure S6).

Although prior studies indicated that AMPK may promote NCOA4 phosphorylation,^31^ ceritinib-induced AMPK activation did not change NCOA4 phosphorylation levels in either cell line (Figure S7), which suggests that AMPK does not directly regulate NCOA4 through phosphorylation under these experimental conditions. Next, we employed the AMPK inhibitor Dorsomorphin to evaluate the functional role of AMPK in ceritinib-triggered ferroptosis. Dorsomorphin effectively blocked ceritinib-induced AMPK phosphorylation (Figure 5B) and partially rescued the decline in cell viability caused by ceritinib (Figure 5C). Mechanistically, AMPK inhibition reversed ceritinib-mediated alterations in key ferroptosis and autophagy regulators: the downregulation of GPX4 and FTH1 was alleviated, and the upregulation of LC3II and ACSL4 was suppressed (Figure 5D). Consistently, biochemical assays demonstrated that Dorsomorphin significantly reduced ceritinib-induced accumulation of LPO, MDA, and intracellular Fe^2+^ (Figures 5E–5G), confirming the requirement of AMPK activation for ferroptotic execution. Moreover, clonogenic assays revealed that AMPK inhibition partially restored ceritinib-suppressed cell proliferation (Figure 5H). In addition, knockdown of AMPK also mitigated ferroptosis triggered by ceritinib (Figures S8A–S8C). Conversely, pharmacological activation of AMPK by MK8722 (7 μΜ, 24h) increased NCOA4 expression, reduced GPX4 levels, and promoted Fe^2+^ accumulation and LPO accumulation (Figures S9A–S9C), indicating that AMPK activation alone is sufficient to drive ferroptosis-associated changes in this context.

To further define the functional consequences of ceritinib-induced AMPK activation, we next investigated the impact of ceritinib on downstream AMPK signaling pathways that govern cellular metabolism and autophagy. Ceritinib decreased mTOR phosphorylation while increasing ACC and ULK1 phosphorylation in both cell lines, indicating activation of the AMPK-ULK1 axis and engagement of metabolic and autophagy-related signaling pathways (Figure S10).

Collectively, these findings delineate a mechanistic pathway in which ceritinib disrupts glucose metabolism via GLUT1 downregulation, inducing energy stress that activates AMPK. Activated AMPK then promotes ferritinophagy and iron dysregulation, thereby driving ferroptosis. Importantly, pharmacological inhibition of AMPK mitigates these effects, establishing AMPK as a central mediator of ceritinib-induced ferroptotic cell death.

### GLUT1 is essential for ceritinib-mediated tumor suppression via AMPK-dependent ferroptosis *in vivo*

**Figure 6:**
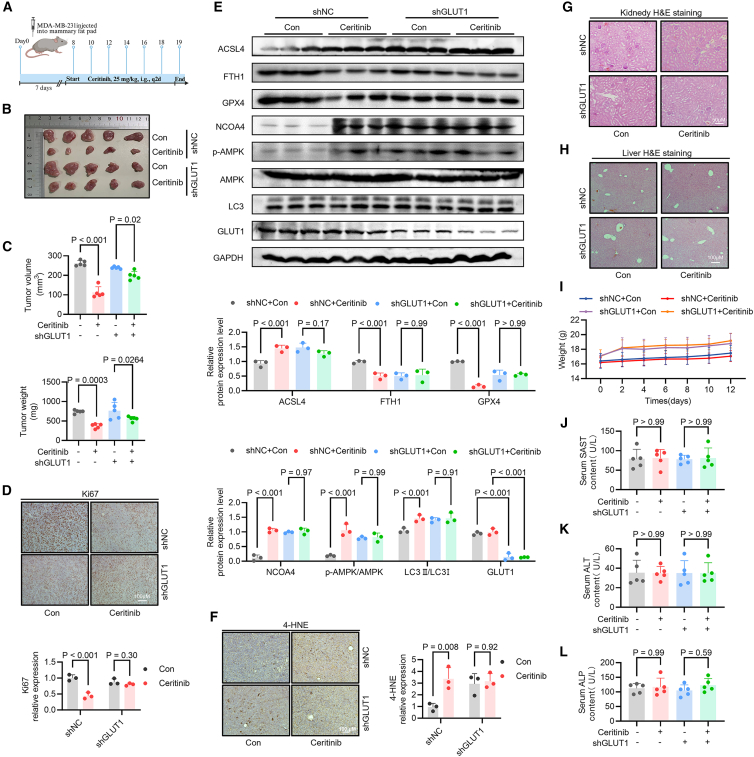
GLUT1 knockdown reverses the tumor-suppressive effects of CEB *in vivo* (A) Schematic overview of the *in vivo* xenograft and treatment workflow. (B) Representative images of tumors excised from mice xenografted with different treatment groups: control (Con), CEB-treated (ceritinib), GLUT1 knockdown (shGLUT1), and CEB-treated with GLUT1 knockdown (ceritinib + shGLUT1), as indicated (*n* = 5). (C) Quantification of tumor volume (top) and tumor weight (bottom) across the four groups (*n* = 5). (D) Immunohistochemical staining and quantitative analysis of of Ki67 in tumor tissues from each treatment group. Scale bars, 100 μM (*n* = 3). (E) Western blot analysis showed the expression of ACSL4, FTH1, GPX4, NCOA4, *p*-AMPK, AMPK, LC3, and GLUT1. (*n* = 3). (F) Immunohistochemical analysis of 4-hydroxynonenal (4-HNE) staining in tumor tissues derived from control (shNC) or GLUT1-knockdown (shGLUT1) MDA-MB-231 xenografts treated with vehicle (Con) or ceritinib. Scale bars, 100 μM (*n* = 3). (G) Hematoxylin and eosin (HE) staining of kidney sections from mice treated with vehicle or ceritinib. Scale bars, 50 μM (*n* = 3). (H) HE staining of liver sections from mice treated with vehicle or ceritinib. Scale bars, 100 μM (*n* = 3). (I) Body weight changes of tumor-bearing mice during ceritinib treatment (*n* = 5). (J–L) Analysis of liver function indicators. Serum levels of (J) AST, (K) ALT, and (L) ALP were measured at the end of the treatment period in mice (*n* = 3). Significance was assessed by one-way ANOVA or two-way ANOVA and Tukey’s post hoc test. The data are shown as the mean ± SEM.

To validate the functional relevance of GLUT1 in ceritinib’s antitumor activity *in vivo*, we established an MDA-MB-231 xenograft model using cells stably expressing either control shRNA (shNC) or GLUT1-targeting shRNA (shGLUT1). Treatment with ceritinib (25 mg/kg, 14 days) robustly inhibited tumor growth in shNC-bearing mice, as reflected by reductions in tumor volume and weight. In contrast, GLUT1 knockdown markedly attenuated the antitumor effect of ceritinib (Figures 6A–6C), demonstrating that GLUT1 is critical for mediating ceritinib’s efficacy *in vivo*. Consistent with these phenotypic outcomes, immunohistochemical analysis of Ki67 revealed that ceritinib-induced suppression of tumor cell proliferation was substantially diminished in shGLUT1 tumors (Figure 6D). At the molecular level, ceritinib treatment in shNC tumors enhanced AMPK activation (elevated *p*-AMPK) and upregulated ferroptosis-associated proteins, including ACSL4 and NCOA4, while downregulating GPX4 and FTH1 (Figure 6E). Notably, GLUT1 knockdown reversed these effects, indicating that GLUT1 is required for ceritinib-induced AMPK activation and subsequent ferroptosis *in vivo*. Lipid peroxidation in tumor tissues was assessed by 4-HNE immunohistochemistry. Ceritinib markedly increased 4-HNE accumulation in shNC tumors, whereas GLUT1 knockdown resulted in low basal 4-HNE levels and abolished ceritinib induced lipid peroxidation (Figure 6F).

Consistently, H&E staining of liver and kidney sections revealed no apparent histopathological abnormalities, indicating the absence of detectable hepatic or renal toxicity (Figures 6G and 6H). Furthermore, ceritinib-treated mice exhibited no significant changes in body weight or serum biochemical parameters, including ALT, AST, and ALP (Figures 6I and 6J–6L). Together, these findings establish GLUT1 as a pivotal mediator of ceritinib’s antitumor activity, linking metabolic regulation to AMPK-dependent ferritinophagy and ferroptosis, and highlighting GLUT1 as a potential target to modulate therapeutic response.

### Ceritinib downregulates GLUT1 via TRIM21-mediated ubiquitination and proteasomal degradation

**Figure 7:**
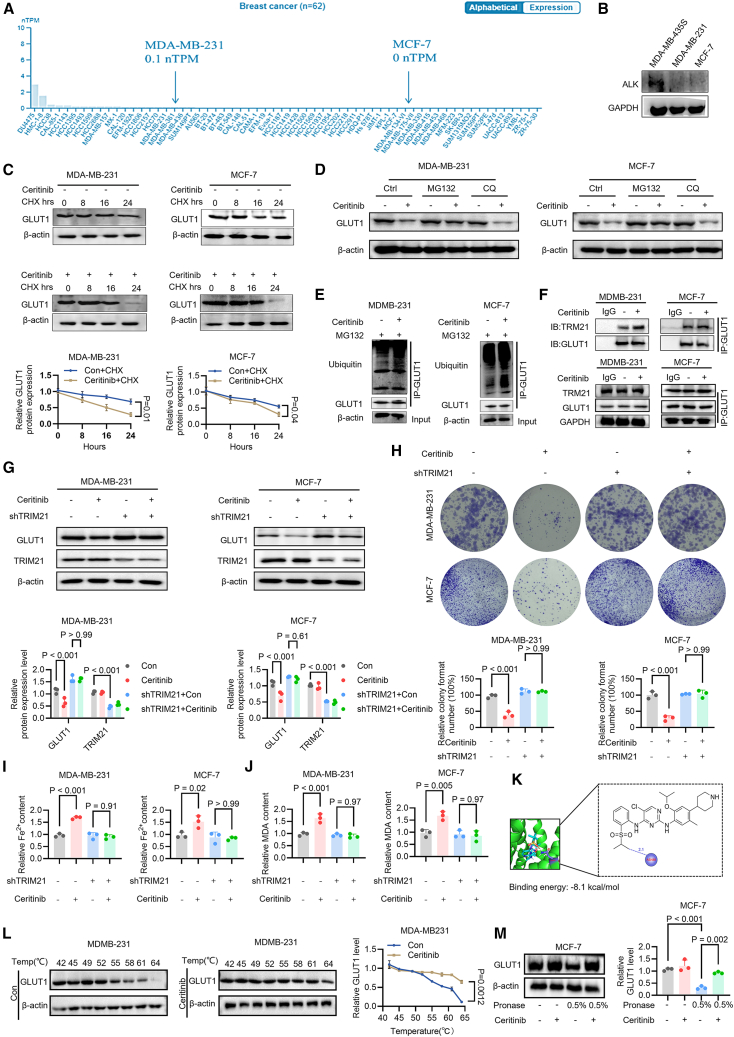
Ceritinib binds to GLUT1 and promotes its ubiquitination (A) Expression profile of ALK in MDA-MB-231 and MCF-7 breast cancer cell lines from the human protein atlas database. (B) Western blot analysis of ALK expression in MDA-MB-231, MDA-MB-435S, and MCF-7 cells (*n* = 3). (C) Western blot showing GLUT1 degradation in MDA-MB-231 and MCF-7 cells treated with ceritinib and CHX over different time points (*n* = 3). (D) MDA-MB-231 and MCF-7 cells were treated with or without ceritinib, following with MG132 or CQ treatment. Level of GLUT1 was determined by western blot (*n* = 3). (E) CoIP analysis of ubiquitination of GLUT1 in MDA-MB-231 and MCF-7 cells treated with ceritinib and MG132 (*n* = 3). (F) CoIP analysis of the interaction between GLUT1 and TRIM21 in MDA-MB-231 and MCF-7 cells treated with ceritinib (*n* = 3). (G) Western blot analysis showed the expression of GLUT1 and TRIM21 levels in MDA-MB-231 and MCF-7 cells treated with ceritinib or control, with or without shTRIM21. (H) Representative images and quantification of colony formation in MDA-MB-231 and MCF-7 cells treated with ceritinib and/or shTRIM21 (*n* = 3). (I) Relative Fe^2+^ content analysis in MDA-MB-231 and MCF-7 cells treated with ceritinib and/or shTRIM21 (*n* = 3). (J) Relative MDA content analysis in MDA-MB-231 and MCF-7 cells treated with ceritinib and/or shTRIM21 (*n* = 3). (K) Molecular docking of ceritinib with GLUT1 (binding energy, −8.1 kcal/mol). (L) CETSA of GLUT1 in MDA-MB-231 cells with and without ceritinib treatment, showing changes in GLUT1 stability at different temperatures (*n* = 3). (M) Cell lysates were subjected to DARTS analysis using 0.5% pronase in MCF-7 cell, followed by Western blot for GLUT1 (*n* = 3). Significance was assessed by one-way ANOVA or two-way ANOVA and Tukey’s post hoc test. The data are shown as the mean ± SEM. Significance was assessed by Student’s *t* test. The data are shown as the mean ± SEM.

To delineate the molecular targets through which ceritinib exerts its effects in breast cancer cells, we first assessed ALK expression. Although ceritinib is a clinically approved ALK inhibitor for NSCLC,^22^ ALK protein was undetectable in both MCF-7 and MDA-MB-231 cells (Figures 7A and 7B), indicating that its antitumor activity in these models is ALK-independent. Building on our findings that GLUT1 downregulation mediates ceritinib-induced ferroptosis, we investigated the mechanism underlying this effect. Ceritinib treatment did not alter GLUT1 mRNA levels (Figure S1B), suggesting post-transcriptional regulation. Assessment of GLUT1 protein stability using cycloheximide (CHX) revealed accelerated degradation in ceritinib-treated cells (Figure 7C), indicating reduced protein half-life. Proteasome inhibition with MG132, but not lysosome inhibition with chloroquine (CQ), blocked ceritinib-induced GLUT1 reduction (Figure 7D), implicating the ubiquitin-proteasome system. Given that TRIM21 is a well-characterized E3 ubiquitin ligase known to target GLUT1 in other cellular contexts,^32^^,^^33^ we examined its role. Ceritinib treatment enhanced the interaction between GLUT1 and TRIM21 (Figures 7E and 7F), suggesting that ceritinib facilitates TRIM21-mediated ubiquitination of GLUT1. To further establish the functional requirement of TRIM21 in this process, we performed genetic loss-of-function experiments. Notably, shRNA-mediated TRIM21 knockdown largely abolished ceritinib induced GLUT1 protein degradation in both breast cancer cell lines (Figure 7G). Importantly, TRIM21 knockdown significantly attenuated the inhibitory effect of ceritinib on clonogenic growth and markedly reduced ceritinib-induced ferroptosis, as reflected by decreased intracellular Fe^2+^ level and MDA level (Figures 7H–7J). These results demonstrate that TRIM21 is indispensable for ceritinib-induced GLUT1 degradation and subsequent ferroptotic cell death. To probe the mechanism further, molecular docking predicted a high-affinity interaction (−8.5 kcal/mol) between ceritinib and GLUT1’s transmembrane domain (Figure 7K), indicating potential direct binding. Supporting this, cellular thermal shift assay (CETSA) showed increased thermal stability of GLUT1 upon ceritinib treatment (Figure 7L), and drug affinity responsive target stability (DARTS) assay confirmed protection of GLUT1 from protease digestion (Figure 7M). Based on this observation, we further detected whether ceritinib could directly target AMPK. CETSA and DARTS assays showed that ceritinib did not alter AMPK thermal stability or protease sensitivity, indicating that there is no direct binding (Figures S11A and S11B). Furthermore, following GLUT1 knockdown, ceritinib failed to further enhance AMPK activation, suggesting that its effect depends on GLUT1 (Figure S11C). Together, these data indicate that ceritinib directly binds to GLUT1, promoting its recognition by TRIM21, leading to ubiquitination and proteasomal degradation. This post-translational downregulation of GLUT1 provides a mechanistic basis for ceritinib-induced metabolic perturbation and ferroptotic cell death in breast cancer cells.

## Discussion

Recent studies have identified that ceritinib’s efficacy may extend beyond its canonical inhibition of ALK, making it a versatile candidate for targeting metabolic vulnerabilities in cancer cells. In our study, we establish a coherent mechanism wherein ceritinib directly binds GLUT1, likely inducing conformational changes that facilitate TRIM21 recognition, thereby triggering ubiquitin-dependent proteasomal degradation. Ceritinib’s off-target effects on metabolism have been suggested in previous studies.^34^^,^^35^ Meanwhile, crizotinib, another ALK inhibitor, also demonstrates the ability to regulate glucose metabolism and autophagy in cancers, suggesting that ALK inhibitors may have broader impacts on tumor biology beyond their intended targets.^36^ These studies and our findings substantiate that ALK inhibitors, particularly ceritinib, hold promise as metabolic modulators for cancer therapy.

Cancer cells proliferate rapidly, driving an increased demand for energy. To meet this demand, GLUT1, a key membrane transporter, mediates basal glucose uptake in most cancers.^37^ Upregulated GLUT1 expression ensures glucose entry into metabolic pathways, supplying the energy required for cancer cell proliferation.^38^ Elevated GLUT1 levels play a vital role in colorectal cancer by facilitating glucose transport and supporting tumor proliferation,^39^ while in esophageal cancer, increased GLUT1 mRNA and protein expression highlights its critical function in enhancing glucose uptake.^40^ Importantly, studies have demonstrated that inhibiting GLUT1 disrupts glucose metabolism, leading to ferroptosis characterized by lipid peroxidation and iron accumulation.^41^ This aligns with our findings where ceritinib-induced GLUT1 degradation leads to ferroptosis in breast cancer cells. Emerging evidence demonstrates that that certain tumor cells exhibit marked susceptibility to ferroptosis, and induction of this cell death mechanism has shown significant anti-tumor efficacy in suppressing tumor progression.^5^^,^^8^^,^^42^ Pharmacological activation of ferroptosis has been consistently shown to suppress malignant cell proliferation in diverse preclinical models, as demonstrated by multiple compound-based investigations.^43^ The identification of ceritinib as a novel GLUT1 stability modulator capable of triggering ferroptosis provides a novel therapeutic strategy for targeting metabolic reprogramming in cancers that rely on GLUT1 for sustained glycolysis.

Furthermore, we found that the inhibition of GLUT1 led to a significant activation of AMPK, which is a heterotrimeric serine/threonine kinase composed of catalytic α, β, and γ subunit, functions as a critical energy sensor that monitors intracellular AMP/ATP ratios. Under energy stress, AMPK activation reprograms cellular metabolism and determines cell fate decisions.^44^ Notably, glucose deprivation activates AMPK through both AMP-dependent and AMP-independent mechanisms, initiating adaptive responses that may promote short-term survival.^45^^,^^46^ Conversely, AMPK is reported to negatively regulate the Warburg effect, promotes a metabolic shift during cancer, and acts as a tumor suppressor that inhibits the energy metabolism, growth and metastasis of tumors.^47^^,^^48^^,^^49^ Consequently, AMPK’s dual roles in oncology are governed by microenvironmental cues. Additionally, emerging evidence suggests that AMPK activation promotes ferroptosis by inducing autophagy and ROS accumulation through inhibition of mTOR and AKT signaling.^50^ Consistent with these studies, our data demonstrated that ceritinib-mediated GLUT1 degradation induced metabolic stress and triggers AMPK activation as an early event, thereby linking glucose restriction to ferroptotic susceptibility.

Previous studies have shown that in esophageal cancer, GLUT1 knockdown activates autophagy and promotes ferroptosis.^41^ Significantly, our study reveals that ceritinib-induced GLUT1 downregulation similarly elicits dual effects-promoting ferroptosis while concomitantly activating autophagy. Moreover, we attribute these coordinated responses to AMPK pathway activation. These findings align with emerging consensus that ferroptosis represents an autophagy-dependent cell death modality.^51^ Mechanistically, ferroptosis requires iron-driven oxidative damage via Fenton chemistry,^52^^,^^53^ where ferritinophagy (NCOA4-mediated autophagic degradation of ferritin) liberates catalytic iron pools.^54^ Our investigations demonstrated a marked upregulation of NCOA4 in ceritinib-treated cells, with elevated ferroptosis levels correlating with NCOA4 upregulation. Thus, ceritinib-induced AMPK activation initiates a coordinated cascade of autophagy induction, ferritinophagy, iron overload, and ferroptotic demise.^55^

The discovery of ceritinib-induced TRIM21-GLUT1 interaction unveils a novel mechanism underlying ceritinib’s modulation of cellular functions. As an E3 ubiquitin ligase, TRIM21 orchestrates ubiquitin-dependent degradation of key regulatory proteins involved in stress responses and metabolic pathways.^33^^,^^56^ Our data demonstrated that ceritinib significantly enhanced TRIM21 binding to GLUT1, ultimately triggering proteasomal degradation of this critical glucose transporter. We propose that ceritinib-induced conformational changes in GLUT1 may expose otherwise cryptic degron motifs, facilitating TRIM21 recognition and ubiquitination, a mechanism analogous to ligand-induced substrate sensitization reported for other TRIM21 targets.^57^^,^^58^ Notably, as GLUT1 conformation shifts dynamically during its transport cycle, ceritinib might stabilize a ubiquitination-permissive state favoring TRIM21 docking. However, further analyses are warranted, including visualizing ceritinib-induced conformational dynamics of GLUT1 and its TRIM21-binding interface through cryo-EM/HDX-MS, mapping TRIM21-mediated ubiquitination sites on GLUT1 to elucidate degradation specificity, and assessing ferroptosis sensitivity using ubiquitination-resistant GLUT1 mutants to establish causal degradation effects. Taken together, this novel interaction represents a key step in ceritinib’s ability to modulate glucose metabolism and induce ferroptosis, making TRIM21 a potential therapeutic target for enhancing ceritinib’s anticancer effects.

In summary, our study reveals that ceritinib exerts its anticancer effects in an ALK-independent manner by directly binding to GLUT1, promoting its TRIM21-mediated ubiquitination and proteasomal degradation. This disruption of glucose metabolism, coupled with the induction of ferritinophagy and ferroptosis, positions ceritinib as a promising metabolic intervention for GLUT1-dependent cancers. These findings not only expand the therapeutic potential of ceritinib beyond its original use as an ALK inhibitor but also highlight the broader applicability of targeting cancer metabolic vulnerabilities. Future studies exploring whether TRIM21-dependent regulation of nutrient transporters represents a common mechanism among kinase inhibitors may further advance drug repurposing strategies in cancer metabolism-oriented therapies.

### Limitations of the study

Despite providing mechanistic insights into ceritinib-mediated regulation of GLUT1 and ferroptosis, several limitations of this study should be acknowledged. First, although our data support a direct interaction between ceritinib and GLUT1, the precise structural basis of this binding and the associated conformational changes remain unresolved. High-resolution structural analyses, such as cryo-electron microscopy or hydrogen-deuterium exchange mass spectrometry, are required to validate the proposed mechanism of ligand-induced exposure of TRIM21-recognition motifs. Second, while we demonstrate that TRIM21 mediates GLUT1 ubiquitination and degradation, the exact ubiquitination sites on GLUT1 and their functional relevance have not been systematically mapped. Mutagenesis studies using ubiquitination-deficient GLUT1 variants would be necessary to establish causality more rigorously.

Finally, the generalizability of our findings across different cancer types remains to be determined, as tumor dependency on GLUT1 and susceptibility to ferroptosis can vary substantially. Addressing these limitations in future studies will be critical for validating the proposed mechanism and for advancing ceritinib as a therapeutic strategy targeting metabolic vulnerabilities in cancer.

## Resource availability

### Lead contact

Further information and requests for resources and reagents should be directed to and will be fulfilled by the lead contact, Zhenbo Song (songzb484@nenu.edu.cn).

### Materials availability

This study did not generate new unique reagents.

### Data and code availability

• No high-throughput sequencing data were generated in this study. All data supporting the findings of this study are available from the corresponding author upon reasonable request.
• This article does not report any original code.
• Any additional information required to reanalyze the data reported in this study is available from the lead contact upon request.

## Acknowledgments

This study was supported by grants from the Research Foundation of Jilin Provincial Science and Technology Development (20240101265JC).

## Author contributions

Y.C.: writing – original draft, methodology, investigation, formal analysis, data curation, and conceptualization; C.L. and R.Z.: methodology, investigation, formal analysis, and data curation; J.L. and Y.L.: formal analysis and data curation; Z.Z. and J.Z.: data curation and formal analysis; F.G.: data curation and funding acquisition; Y.B., L.S. and S.W.: data curation; L.Z., Y.S. and G.W.: investigation; J.L., X.Y. and Z.S.: supervision, project administration, conceptualization, and writing – review and editing.

## Declaration of interests

The authors declare that they have no conflict of interest.

## STAR★Methods

### Key resources table

**Table:** 

REAGENT or RESOURCE	SOURCE	IDENTIFIER
**Antibodies**		

Anti-ACSL4/FACL4	Proteintech	Cat#22401-1-AP; RRID: N/A
Anti-AIFM2/FSP1	Proteintech	Cat#20886-1-AP; RRID: N/A
Anti-GPX4	Abcam	Cat#ab125066; RRID: AB_10973901
Anti-Keap1	Abcam	Cat#ab119403; RRID: AB_10902777
Anti-Nrf2	Abcam	Cat#ab62352; RRID: AB_944418
Anti-SLC7A11	Abcam	Cat#ab151751; RRID: AB_2714083
Anti-GLUT1	Proteintech	Cat#21829-1-AP; RRID: N/A
Anti-Hexokinase 2	Proteintech	Cat#22029-1-AP; RRID: N/A
Anti-PGAM1	Proteintech	Cat#16126-1-AP; RRID: N/A
Anti-LDHA	Proteintech	Cat#19987-1-AP; RRID: N/A
Anti-PKM2	Proteintech	Cat#15822-1-AP; RRID: N/A
Anti-LC3B	Cell Signaling Technology	Cat#14600; RRID: AB_2137707
Anti-NCOA4	Proteintech	Cat#83394-4-RR; RRID: N/A
Anti-Ferritin Heavy Chain (FTH1)	Abcam	Cat#ab83428; RRID: AB_10673707
Anti-p62/SQSTM1	Proteintech	Cat#18420-1-AP; RRID: N/A
Anti-Beclin1	Proteintech	Cat#11306-1-AP; RRID: N/A
Anti-OGDH	Proteintech	Cat#15212-1-AP; RRID: N/A
Anti-PDHE1	Proteintech	Cat#8068-1-AP; RRID: N/A
Anti-IDH2	Proteintech	Cat#82117-1-RR; RRID: N/A
Anti-ALK	Wanlei Biological	Cat#WL05530; RRID: N/A
Anti-Ubiquitin	Proteintech	Cat#10201-2-AP; RRID: N/A
Anti-AMPKα	Proteintech	Cat#10929-2-AP; RRID: N/A
Anti-phospho-AMPKα	Proteintech	Cat#80209-6-RR; RRID: N/A
Anti-COX2/PTGS2	Proteintech	Cat#27308-1-AP; RRID: N/A
Anti-TRIM21	HUABIO	Cat#HA721832; RRID: N/A
Anti-mTOR	Cell Signaling Technology	Cat#2983; RRID: AB_2105622
Anti-phospho-mTOR (Ser2448)	Cell Signaling Technology	Cat#5536; RRID: AB_10691552
Anti-Acetyl-CoA Carboxylase (ACC)	Cell Signaling Technology	Cat#3676; RRID: AB_2219400
Anti-phospho-ACC (Ser79)	Cell Signaling Technology	Cat#11818; RRID: AB_2687530
Anti-ULK1	Zenbio	Cat#R381887; RRID: N/A
Anti-phospho-ULK1	Zenbio	Cat#R30281; RRID: N/A
Anti-Pan-Phospho-Ser/Thr	Cell Signaling Technology	Cat#9631; RRID: AB_330830
Normal Rabbit IgG	Proteintech	Cat#30000-0-AP; RRID: N/A
Anti-GAPDH	Proteintech	Cat#60004-1-Ig; RRID: N/A
Anti-β-actin	Proteintech	Cat#66009-1-Ig; RRID: N/A
HRP Goat Anti-Mouse IgG (H+L)	Proteintech	Cat#SA00001-1; RRID: N/A
HRP Goat Anti-Rabbit IgG (H+L)	Proteintech	Cat#SA00001-2; RRID: N/A
Mouse Anti-Rabbit IgG HRP	Abmart	Cat#M21008; RRID: N/A
Ki67	Proteintech	Cat#27309-1-AP; RRID: N/A
4-Hydroxynonenal	Proteintech	Cat#68538-1-Ig; RRID: N/A

**Chemicals, peptides, and recombinant proteins**		

Ceritinib	MedChemExpress	Cat# HY-15656
Ferrostatin-1 (Fer-1)	MedChemExpress	Cat# HY-100579
3-Methyladenine (3-MA)	MedChemExpress	Cat# HY-19312
MK8722	MedChemExpress	Cat# HY-111363
Necrostatin-1 (Nec-1)	MedChemExpress	Cat# HY-15760
Z-VAD-FMK	MedChemExpress	Cat# HY-16658B
Dorsomorphin	MedChemExpress	Cat# HY-13418A
Cycloheximide (CHX)	MedChemExpress	Cat# HY-12320
Fetal bovine serum (FBS)	ExCell Bio	Cat# FSP500
RPMI 1640 medium	Gibco	Cat# 11875093
Penicillin-streptomycin	Gibco	Cat# 15140122
Trypsin-EDTA (0.25%)	Solaibio	Cat# T1300
Methanol	Beijing Chemical Reagents Company	Cat# N/A
Ethanol	Beijing Chemical Reagents Company	Cat# N/A
Isopropanol	Beijing Chemical Reagents Company	Cat# N/A
MTT	Beyotime	Cat#ST316
Lipofectamine 2000	Thermo Fisher Scientific	Cat# 11668027
Opti-MEM	Gibco	Cat# 31985070
Trizol	Thermo Fisher Scientific	Cat# 15596026CN
2 × Q5 SYBR qPCR Master Mix (Universal)	TOLOBIO	Cat# 22208
Trypan Blue Staining Solution	Beyotime	Cat# C1313S
Dimethyl sulfoxide (DMSO)	Beyotime	Cat# ST038
Crystal violet	Beyotime	Cat# Y268090
FerroOrange	Dojindo	Cat# F374
Pronase	Sigma-Aldrich	Cat# 53702
Protein A/G magnetic beads	MedChemExpress	Cat# HY-K0202
Protein Molecular Weight Marker	SparkJade	Cat# EC1020
PBS	Servicebio	Cat# G4202
BSA	Servicebio	Cat# GC305006
4% Paraformaldehyde Fix Solution	Beyotime	Cat# P0099
Triton X-100	Beyotime	Cat# P0096-
PVDF Membrane	Thermo Fisher Scientific	Cat# 88518
ECL	BOSTER	Cat# AR1191
Protease inhibitor/phosphatase inhibitor	Beyotime	Cat# P1045
Cell lysis buffer for Western and IP	Beyotime	Cat# P0013
RIPA lysis buffer	Beyotime	Cat# P0013E

**Critical commercial assays**		

BCA Protein Assay Kit	Beyotime	Cat# P0012
SDS-PAGE Gel Kit	Servicebio	Cat# G2037
Glucose Assay Kit	Nanjing Jiancheng Bioengineering Institute	Cat# F006-1-1
Pyruvate Assay Kit	Solarbio	Cat# A081-1-1
Lipid Peroxidation Assay Kit	Nanjing Jiancheng Bioengineering Institute	Cat# A106-1-1
MDA Assay Kit	Nanjing Jiancheng Bioengineering Institute	Cat# A003-4-1
Iron Assay Kit	Nanjing Jiancheng Bioengineering Institute	Cat# A039-2-1
NADP^+^/NADPH Assay Kit with WST-8	Beyotime	Cat# S0179
GSH and GSSG Assay Kit	Beyotime	Cat# S0053
GPX4 Activity Assay Kit	Solarbio	Cat# BC6260
AST ELISA Kit	COIBO BIO	Cat# CB10659-Mu
ALT ELISA Kit	COIBO BIO	Cat# CB15201-Mu
ALP ELISA Kit	COIBO BIO	Cat# CB10405-Mu
ROS Assay Kit (DCFH-DA)	Beyotime	Cat# S0033S
Mitochondrial Complex I Activity Assay Kit	Solarbio	Cat# BC0515
Mitochondrial Complex III Activity Assay Kit	Solarbio	Cat# BC3240
Mitochondrial Complex V Activity Assay Kit	Solarbio	Cat# BC1440
Mammalian Genomic DNA Extraction Kit	Beyotime	Cat# D0061
FITC Annexin V Apoptosis Detection Kit I	BD Biosciences	Cat# 556547
ToloScript RT EasyMix for qPCR (with 2-Step gDNA Erase-Out)	TOLOBIO	Cat# 22106

**Experimental models: cell lines**		

MCF-7 human breast cancer cell line	ATCC	HTB-22; RRID: CVCL_0031
MDA-MB-231 human breast cancer cell line	ATCC	HTB-26; RRID: CVCL_0062
MDA-MB-435S human cell line	ATCC	HTB-129; RRID: CVCL_0622

**Experimental models: organisms/strains**		

Female BALB/c Nude mice	Beijing Vital River Laboratory Animal Technology Co., Ltd	N/A

**Recombinant DNA**		

pLV3-CMV-SLC2A1(human)-3×FLAG-CopGFP-Puro plasmid	MIAOLING PLASMID	Cat# P54238
pLV3-U6-SLC2A1(human)-shRNA-CopGFP-Puro plasmid	MIAOLING PLASMID	Cat# P71068
pLV3-U6-TRIM21(human)-shRNA1-CopGFP-Puro plasmid	MIAOLING PLASMID	Cat# P88480
pSuper-Puro-AMPK-shRNA(human) plasmid	MIAOLING PLASMID	Cat# P18867

**Oligonucleotides**		

RT-qPCR primers for detection of expression of GLUT1, NCOA4, FTH1, β-actin, ND1and B2M See Table S1 in supplemental information	This Paper	N/A

**Software and algorithms**		

GraphPad Prism software (version 8.3.0)	GraphPad	N/A
ImageJ software	NIH	N/A
AutoDock4	Scripps	N/A
Primer (version 5.0)	Premier	N/A

**Other**		

LSM 980	ZEISS	N/A
OLYMPUS	Olympus Corporation	N/A

### Experimental model and study participant details

#### Human participants

This study did not involve human participants or human-derived samples.

#### Animal models

All animal experiments were approved by the Institutional Animal Care and Use Committee (NENU/IACUC, AP20241210, Approval date 10 December 2024) at Northeast Normal University, China. All procedures were conducted in accordance with relevant institutional guidelines and regulations. Only female mice were used in this study due to the female-dominant nature of breast cancer. Consequently, the potential influence of sex on the findings could not be evaluated, which we acknowledge as a limitation. Gender is not applicable to this animal study.

#### Cell lines

Human melanoma cell line MDA-MB-435S, along with human breast cancer cell lines MDA-MB-231 and MCF-7, were sourced from the American Type Culture Collection (ATCC, Virginia, USA). Both cell lines were routinely tested and confirmed to be mycoplasma-negative and were authenticated by short tandem repeat (STR) profiling prior to use.

### Method details

#### Reagents

Ceritinib (HY-15656), Ferrostatin-1 (Fer-1, HY-100579), 3-methyladenine (3-MA, HY-19312), MK8722 (HY-111363), Necrostatin-1 (Nec-1, HY-15760), Z-VAD-FMK(HY-16658B), Dorsomorphin (HY-13418A), and Cycloheximide (CHX, HY-12320) were purchased from MedChemExpress (Shanghai, China). Fetal bovine serum (FBS) was obtained from ExCell (Suzhou, China). RPMI 1640 medium, penicillin-streptomycin solution, and trypsin were purchased from Gibco (Grand Island, NY, USA). Analytical-grade chemicals, including methanol, ethanol, and isopropanol, were purchased from Beijing Chemical Reagents Company (Beijing, China).

#### Drug treatment

For drug treatment, ceritinib was used at a final concentration of 8 μM for 24 h in all experiments unless otherwise specified. For experiments requiring alternative treatment conditions, including different concentrations or treatment durations, the specific parameters are explicitly indicated in the corresponding figure legends or method descriptions.

#### Cell culture

Human melanoma cell line MDA-MB-435S, along with human breast cancer cell lines MDA-MB-231 and MCF-7, were sourced from the American Type Culture Collection (ATCC, Virginia, USA). All cells were cultured in RPMI 1640 medium supplemented with 10% FBS and 1% penicillin-streptomycin at 37°C in a humidified incubator containing 5% CO_2_. Subculturing was performed using 0.25% trypsin-EDTA when cells reached 80-90% confluence.

#### Cell protein extraction and western blot analysis

For total protein extraction, cells were washed twice with ice-cold PBS and harvested using a cell scraper. Lysis buffer was then added, and the lysates were incubated on ice for 30 minutes with vortexing every 10 minutes. Subsequently, the samples were centrifuged at 12,000 rpm for 15 minutes at 4°C. The supernatants were collected, and protein concentrations were determined using a BCA protein assay kit. Equal amounts of protein were mixed with loading buffer, denatured at 100°C for 10 minutes, and stored at -80°C until further analysis.

Equal amounts of protein were separated by SDS-PAGE (8-12% gels) and transferred to PVDF membranes. Membranes were blocked with 5% non-fat milk in TBST for 1 h at room temperature, then incubated overnight at 4°C with primary antibodies. After TBST washes, membranes were probed with HRP-conjugated secondary antibodies for 1 h at room temperature. Protein bands were visualized using an enhanced chemiluminescence (ECL) detection system, with GAPDH or β-actin serving as the loading control for normalization.

#### MTT and colony formation assay

Cell viability was measured using the MTT assay. Briefly, cells were harvested with trypsin, neutralized with complete medium, and counted using a hemocytometer. A total of 8 × 10^3^ cells/well were seeded in 96-well plates in 100 μL complete medium, with three replicate wells per condition. After a 24 h incubation at 37°C in a humidified atmosphere with 5% CO_2_, the cells were treated with ceritinib. Following the treatment period, 20 μL of MTT solution (1 mg/mL in PBS) was added to each well and incubated for 4 h at 37°C. The supernatant was then removed, and 100 μL of dimethyl sulfoxide (DMSO) was added to dissolve the formazan crystals. Plates were protected from light and gently shaken for 10 min before measuring absorbance at 570 nm using a microplate reader.

For the colony formation assay, cells were seeded into 6-well plates at a density of 500-1,000 cells per well and incubated in complete medium for 10-14 days to allow colony formation. The medium was refreshed every 3 days. At the endpoint, colonies were washed twice with PBS, fixed with 4% paraformaldehyde for 15 min, and stained with 0.1% crystal violet for 20 min at room temperature. Plates were gently washed with water, air-dried, and the number of colonies containing more than 50 cells was counted manually under a light microscope.

#### Plasmid transfection

The plasmids pLV3-CMV-GLUT1, pLV3-U6-shTRIM21, pSuper-Puro-shAMPK and pLV3-U6-shGLUT1 were obtained from the Miaoling Plasmid Platform (Wuhan, China). Cells were seeded in culture plates at an appropriate density to achieve approximately 50% confluency prior to transfection. One hour before transfection, the culture medium was aspirated and replaced with 200 μL of Opti-MEM reduced-serum medium per well. For transfection complex preparation, 500 ng of plasmid DNA was diluted in 50 μL of Opti-MEM and gently mixed by pipetting 4-5 times. In a separate tube, 2 μL of Lipofectamine 2000 reagent was diluted in 50 μL of Opti-MEM and incubated at room temperature for 5 minutes. The diluted DNA and Lipofectamine solutions were then combined, mixed thoroughly, and incubated for 20-25 minutes to allow complex formation. Subsequently, 100 μL of the transfection mixture was added dropwise to each well, and cells were incubated for 4-6 hours before replacing the medium with fresh complete medium supplemented with 10% fetal bovine serum (FBS). After 48 hours, cells were harvested.

#### Trypan blue exclusion assay

After removing the culture medium, cells were washed with 800 μL PBS to eliminate residual serum and medium. Subsequently, 700 μL of 0.04% (w/v) trypan blue solution was added, and the cells were incubated for 5 minutes under sterile conditions. The staining solution was then discarded, and the cells were gently rinsed with PBS until no visible dye residue remained. Finally, 200 μL of PBS was added, and the cells were examined and imaged under a light microscope. Viable cells excluded the dye and remained unstained, while non-viable cells appeared blue.

#### Annexin V-FITC apoptosis detection assay

Apoptosis was assessed using the FITC Annexin V Apoptosis Detection Kit I according to the manufacturer’s instructions. Cells were seeded in 6-well plates and allowed to adhere overnight. For treatment, the culture medium in the ceritinib group was replaced with fresh medium containing ceritinib and incubated for 24 h. Following treatment, cells were washed twice with cold PBS and resuspended in Binding Buffer at a concentration of 1 × 10^3^ cells/mL. A total of 1 × 10^3^ cells were transferred to a new tube, followed by the addition of 5 μL FITC Annexin V and 5 μL propidium iodide (PI). Samples were incubated for 15 min at room temperature in the dark. Subsequently, 400 μL Binding Buffer was added to each tube, and samples were analyzed by flow cytometry within 1 h. Cells positive for FITC Annexin V and negative for PI were considered apoptotic. Double-positive cells (FITC Annexin and PI) were classified as late apoptotic or necrotic. Cells negative for both markers were regarded as viable.

#### Measurement of lactate production, glucose consumption, and pyruvate content

Cells were seeded into 96-well plates with three replicate wells per group. After cell attachment, the culture medium in the ceritinib treatment group was replaced with medium containing ceritinib and incubated for an additional 24 h. Glucose consumption, lactate production, and pyruvate content were measured using the Glucose Assay Kit, Lactate Assay Kit, and Pyruvate Assay Kit, respectively, according to the manufacturers’ instructions. All measurements were normalized to total protein content.

#### Lipid peroxidation (LPO), malondialdehyde (MDA), and ferrous iron (Fe^2+^) measurement

Cells were seeded in 6-well plates and allowed to adhere. For the ceritinib treatment group, the culture medium was replaced with medium containing ceritinib and incubated for 24 h. LPO, MDA and intracellular Fe^2+^ levels were measured using the Lipid Peroxidation Assay Kit, MDA Assay Kit, and Iron Assay Kit, respectively, following the manufacturers’ protocols. All results were normalized to total protein concentration.

#### Measurement of NADPH level and GSH/GSSG ratio

Cells were seeded in 6-well plates and allowed to adhere. For the ceritinib treatment group, the culture medium was replaced with medium containing ceritinib and incubated for 24 h. The NADPH level and GSH/GSSG ratio were measured using the NADP^+^/NADPH Assay Kit with WST-8 and the GSH and GSSG Assay Kit, respectively, according to the manufacturers’ protocols. All measurements were normalized to total protein concentration.

#### Glutathione peroxidase 4 (GPX4) Activity Assay

Glutathione peroxidase 4 (GPX4) activity was measured using a commercial GPX4 Activity Assay Kit according to the manufacturer’s instructions.

#### Aspartate aminotransferase (AST)/ Alanine aminotransferase (ALT)/Alkaline phosphatase (ALP) activity assay

AST was measured using a commercial AST ELISA Kit according to the manufacturer’s instructions. ALT was measured using a commercial ALT ELISA Kit according to the manufacturer’s instructions. ALP was measured using a commercial ALP ELISA Kit according to the manufacturer’s instructions.

#### Measurement of intracellular reactive oxygen species (ROS)

Cells were seeded in 6-well plates and allowed to adhere. For the ceritinib treatment group, the culture medium was replaced with medium containing ceritinib and incubated for 24 h. After treatment, cells were washed with PBS and incubated with 2′,7′-dichlorofluorescin diacetate at a final concentration of 10 μM for 30 minutes at 37°C in the dark. Subsequently, cells were rinsed with PBS, lysed with appropriate lysis buffer, and the fluorescence intensity of the cell lysates was measured using a fluorescence microplate reader (excitation wavelength: 488 nm; emission wavelength: 525 nm). The ROS levels were normalized to total protein content.

#### Measurement of mitochondrial complex I, III, and V activities

Cells were seeded into 6-well plates and allowed to adhere before the medium in the ceritinib treatment group was replaced with ceritinib-containing medium. The cells were then cultured for 24 h. The enzymatic activities of mitochondrial complexes I, III, and V were quantified using the Mitochondrial Complex I Activity Assay Kit, Mitochondrial Complex III Activity Assay Kit, and Mitochondrial Complex V Activity Assay Kit, respectively, following the manufacturer’s instructions. All values were adjusted based on protein concentrations.

#### Quantitative real-time PCR (qRT-PCR) detection

Primers for qRT-PCR were designed using Primer 5.0 software (Table S1) and synthesized by Sangon Biotech (Shanghai, China). Cells were seeded into 6-well plates and allowed to adhere. After drug treatment, cells were washed with PBS, and total RNA was extracted using Trizol reagent according to the manufacturer’s instructions. Complementary DNA (cDNA) was synthesized using a reverse transcription kit. qRT-PCR was performed using SYBR qPCR Master Mix on a fluorescence-based detection system. β-actin was used as the internal control. Each reaction (20 μL total volume) consisted of 2 μL cDNA (1000 ng/μL), 10 μL SYBR qPCR Master Mix, 1 μL forward primer (500 nmol/L), 1 μL reverse primer (500 nmol/L), and 6 μL RNase-free water. The thermal cycling conditions were as follows: initial denaturation at 95°C for 1 min, followed by 40 cycles of denaturation at 95°C for 15 s, annealing at 60°C for 15 s, and extension at 72°C for 15 s. A melting curve analysis was performed from 60°C to 95°C to verify the specificity of amplification. All reactions were conducted in triplicate, and relative gene expression levels were calculated using the 2^−ΔΔCT^ method.

#### mtDNA copy number detection

Genomic DNA was isolated from the cells using the Mammalian Genomic DNA Extraction Kit. The relative mtDNA copy number was quantified by real-time PCR using primers specific for the ND1 gene, normalized to nuclear DNA B2M content.^59^ The primer sequences used for qRT-PCR are listed in the key resources table (Oligonucleotides). The expression levels of mtDNA were measured in triplicate using the SYBR qPCR Master Mix -based quantitative method. The mtDNA copy number was normalized using the comparative threshold cycle (2^-ΔΔCT^) method.

#### FerroOrange staining and immunofluorescence

Cells were seeded onto glass coverslips placed in 24-well culture plates and allowed to adhere. After drug treatment, 1 μM FerroOrange fluorescent probe was added to the cells, followed by incubation overnight at 37°C in a humidified incubator. The next day, the staining solution was removed, and cells were fixed with 4% paraformaldehyde for 20 minutes at room temperature.

Following fixation, cells were permeabilized with 0.1% Triton X-100 for 10 minutes. To block non-specific binding, cells were incubated with 5% bovine serum albumin (BSA) for 30 minutes at room temperature. Subsequently, cells were stained with DAPI for 10 minutes at room temperature. Fluorescence images were acquired using a laser scanning confocal microscope.

#### Cellular thermal shift assay (CETSA)

Cells were seeded into 10 cm culture dishes and cultured for 24 h. After incubation, cells were rinsed with PBS, harvested, and lysed on ice for 15 minutes in RIPA lysis buffer supplemented with a complete protease inhibitor cocktail. The lysates were then centrifuged at 12,000 × *g* for 15 minutes at 4°C, and the supernatants were collected. The clarified lysates were incubated with 10 μM ceritinib for 1 h at room temperature. Following incubation, the lysates were aliquoted into 50 μL portions and subjected to heat treatment at 42°C, 45°C, 49°C, 52°C, 55°C, 58°C, 61°C, and 64°C for 5 minutes. After heating, samples were immediately centrifuged at 12,000 × *g* for 15 minutes at 4°C to separate the soluble protein fractions. The resulting supernatants were transferred to fresh microcentrifuge tubes and analyzed by Western blot to assess protein thermal stability.

#### Drug affinity responsive target stability (DARTS)

We employed the DARTS assay to identify the protein targets of ceritinib. This label-free method is based on the principle that the stability of target proteins increases upon binding to small molecules, which protects them from proteolysis. First, proteins lysate from cells were prepared by cell lysis buffer. The supernatant was then incubated with ceritinib (10 μM) for 2 h. Following incubation, 0.5% pronase was added for 30 min to degrade unbound proteins. Finally, the identified proteins were validated using Western blot to confirm the interaction.

#### Immunoprecipitation

Cells were lysed on ice using IP lysis buffer supplemented with protease inhibitor/phosphatase inhibitor. Cell lysates were incubated on ice for 30 min and centrifuged at 12,000 × g for 15 min at 4°C to remove debris. Protein concentration was determined using a BCA protein assay kit.

For immunoprecipitation, 2 mg of total protein was incubated with 4 μg of anti-GLUT1/anti-NCOA4 antibody overnight at 4°C with gentle rotation. The next day, 20 μL of Protein A/G magnetic beads were added and incubated for an additional 2-3 h at 4°C. Beads were washed three times with cold IP lysis buffer and once with PBST, followed by elution through boiling in SDS loading buffer at 95°C for 5 min.

The eluted proteins were then subjected to SDS-PAGE and western blot analysis.

#### *In vivo* xenograft model

Female BALB/c nude mice (Vital River, Beijing) were used under IACUC approval (NENU/IACUC AP20241210). MDA-MB-231 shNC or shGLUT1 cells were injected subcutaneously (flank). Cells were suspended in 100 μL of serum-free medium and subcutaneously injected into the right flank of female nude mice. Mice in the shNC group were inoculated with 1×10^6^cells per mouse, whereas mice in the shGLUT1 group were inoculated with 5 × 10^6^cells per mouse. After tumor establishment, mice were randomized into four groups (n = 5): control, ceritinib (25 mg/kg/day by oral gavage), shGLUT1, shGLUT1 + ceritinib. Tumor volumes (V = L × W^2^ × 0.5) were measured every 3 days. After 4 weeks, mice were sacrificed by cervical dislocation, and tumors excised and weighed.

#### Immunohistochemical staining for Ki-67/(4-hydroxynonenal) 4-HNE

Paraffin-embedded tumor tissues were sectioned at 4 μm, deparaffinized, rehydrated, and subjected to antigen retrieval in citrate buffer (pH 6.0). After blocking endogenous peroxidase with 3% H_2_O_2_ and nonspecific binding with 5% BSA, sections were incubated with anti-Ki67/ anti-4-HNE antibody overnight at 4°C, followed by HRP-conjugated secondary antibody. Signals were developed using DAB and counterstained with hematoxylin, dehydrated, mounted, and imaged under a bright-field microscope. Positive nuclei were quantified using ImageJ.

#### Hematoxylin and eosin (H&E) staining

Mouse liver and kidney tissues were fixed in 4% paraformaldehyde at 4°C overnight, dehydrated through a graded ethanol series, cleared in xylene, and embedded in paraffin. Tissue blocks were sectioned at 4 μm thickness. After deparaffinization and rehydration, sections were stained with hematoxylin for 3 min, rinsed in running water, differentiated in 1% acid alcohol, and blued in 0.1% ammonia water. Slides were then counterstained with eosin for 1 min, dehydrated, cleared, and mounted with neutral resin. Histological morphology was examined and imaged under a light microscope (OLYMPUS).

#### Statistical analysis

All experiments were independently repeated at least three times. All data are shown as the mean ± standard error of the mean (SEM). Data were analyzed using IBM SPSS Statistics and GraphPad Prism (version 10.0). For more than two groups, one-way ANOVA with Bonferroni multiple comparisons test was used, whereas for experiments with a second variable, two-way ANOVA with Bonferroni multiple comparisons test was performed. A significance level of P < 0.05 was considered statistically significant.

## Supplemental information

Document S1. Figures S1–S11

Table S1. Primer sequences for qRT-PCR detection
