# Supplementary material for: Ceritinib induces ferroptosis via TRIM21-mediated GLUT1 ubiquitination and AMPK-driven metabolic reprogramming in breast cancer
Source: iScience. 2026 Apr 22;29(6):115846. doi: 10.1016/j.isci.2026.115846 (PMC13195780; doi:10.1016/j.isci.2026.115846)
Supplement: Document S1. Figures S1–S11 [file mmc1.pdf]

iScience, Volume *29*

Supplemental information

Ceritinib induces ferroptosis via TRIM21-mediated GLUT1 ubiquitination and AMPK-driven metabolic reprogramming in breast cancer

Yao Chen, Changchang Liu, Ruonan Zhen, Jiarui Li, Yanran Li, Ziying Zhang, Jingying Zhang, Feng Gao, Yongli Bao, Luguo Sun, Shuyue Wang, Lihua Zheng, Ying Sun, Guannan Wang, Jiawei Li, Xiaoguang Yang, and Zhenbo Song

Supplemental information

Ceritinib Induces Ferroptosis via TRIM21-Mediated GLUT1 Ubiquitination and AMPK-Driven Metabolic Reprogramming in Breast Cancer

*Corresponding author: Zhenbo Song, National Engineering Laboratory for Druggable Gene and Protein Screening, Northeast Normal University, NO.5268 Renmin Street, Changchun 130117, China. E-mail: songzb484@nenu.edu.cn

This PDF file includes:

Fig. S1 to S11

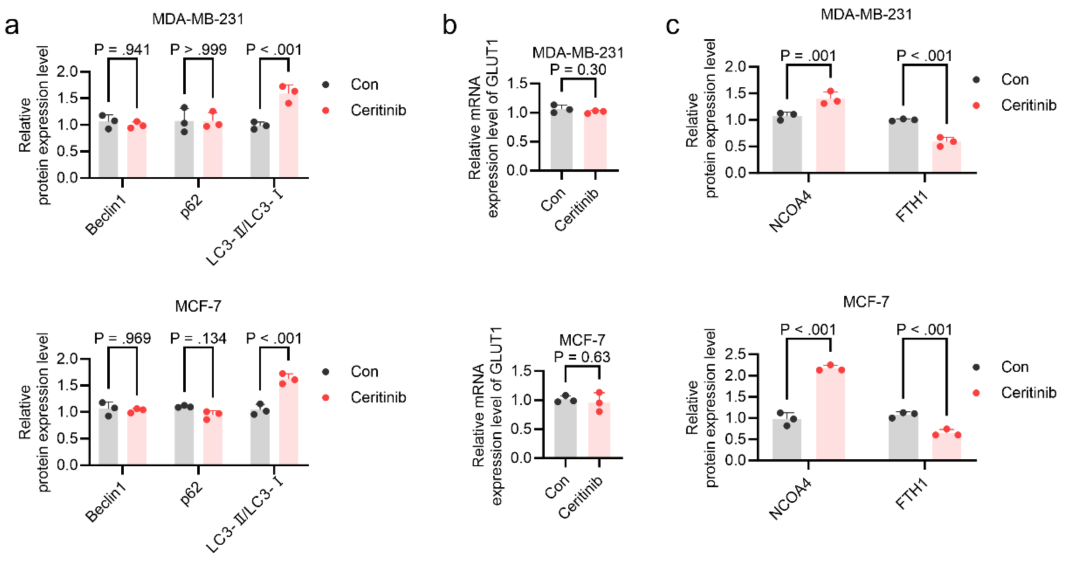

Fig. S1 Ceritinib induces ferritinophagy in breast cancer cells

(a) Western blot quantification analysis showing relative protein expression levels of Beclin1, p62, and LC3II/LC3I in MDA-MB-231 and MCF-7 cells treated with ceritinib compared to the control (n=3).

(b) Relative mRNA expression levels of GLUT1 in MDA-MB-231 and MCF-7 cells after ceritinib treatment (n=3).

(c) Western blot analysis showing relative protein expression levels of NCOA4 and FTH1 in MDA-MB-231 and MCF-7 cells treated with ceritinib compared to the control (n=3).

Significance was assessed by Student's t-test or 2-way ANOVA and Tukey's post hoc test. The data are shown as the mean ± SEM.

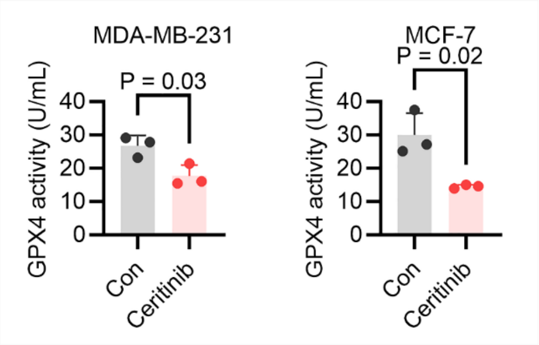

Fig. S2 Ceritinib reduces GPX4 enzymatic activity in breast cancer cells

GPX4 activity levels in MDA-MB-231 and MCF-7 cells following treatment with ceritinib (n = 3).

Significance was assessed by Student's t-test. The data are shown as the mean ± SEM.

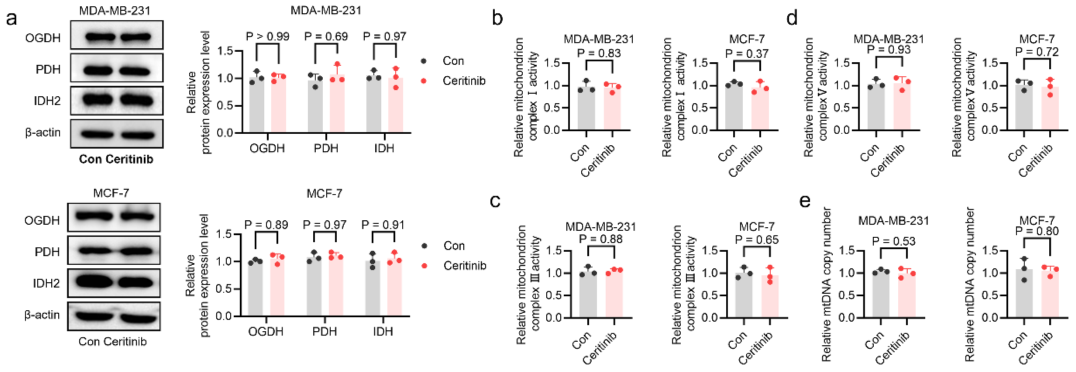

Fig. S3 Ceritinib does not affect mitochondrial metabolism in breast cancer

(a) Western blot analysis of OGDH, PDH, and IDH2 protein expression in MDA-MB-231 and MCF-7 cells treated with or without ceritinib (n = 3).

(b-d) Mitochondrial complex I (b), complex III (c), and complex V (d) activities were measured using enzyme activity assay kits (n = 3).

(e) The relative mitochondrial DNA (mtDNA) copy number was analyzed by qRT-PCR (n = 3).

Data are expressed as the mean ± SEM and were analyzed by Student's t-test, two-way ANOVA, or Tukey's post hoc test where applicable.

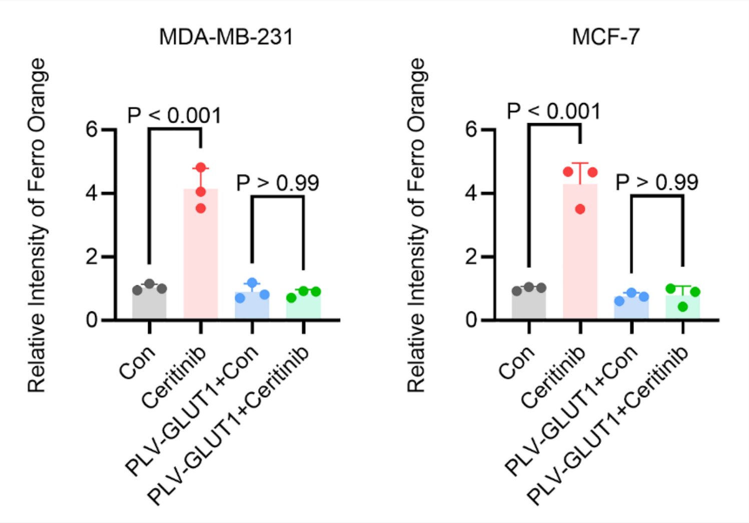

Fig. S4 Ceritinib induces ferroptosis by modulating intracellular Fe^{2+} levels through GLUT1 regulation

Relative intensity of FerroOrange (n=3).

Data are expressed as the mean ± SEM and were analyzed by Student's t-test, two-way ANOVA, or Tukey's post hoc test where applicable

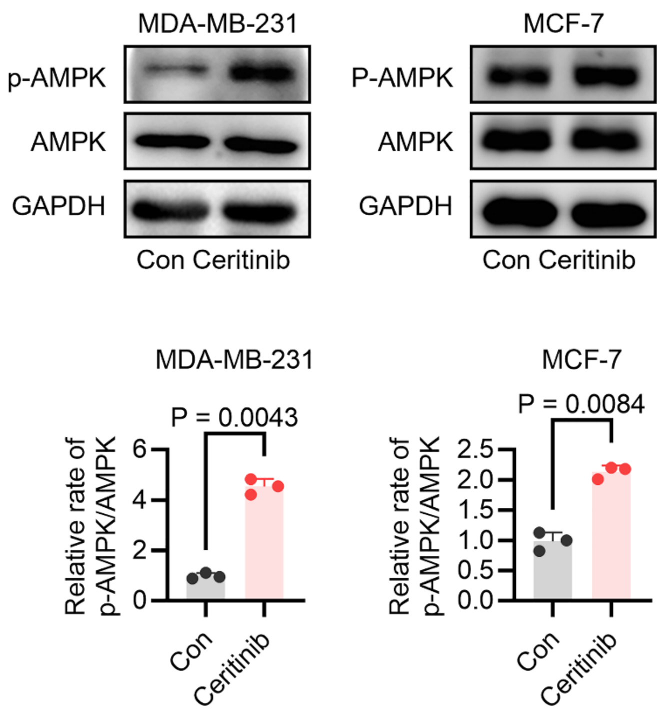

Fig. S5 Ceritinib activates the AMPK signaling pathway in breast cancer

Western blot analysis the expression of p-AMPK and AMPK levels in MDA-MB-231 and MCF-7 cells treated with or without ceritinib (n = 3).

Significance was assessed by Student's t-test. The data are shown as the mean ± SEM.

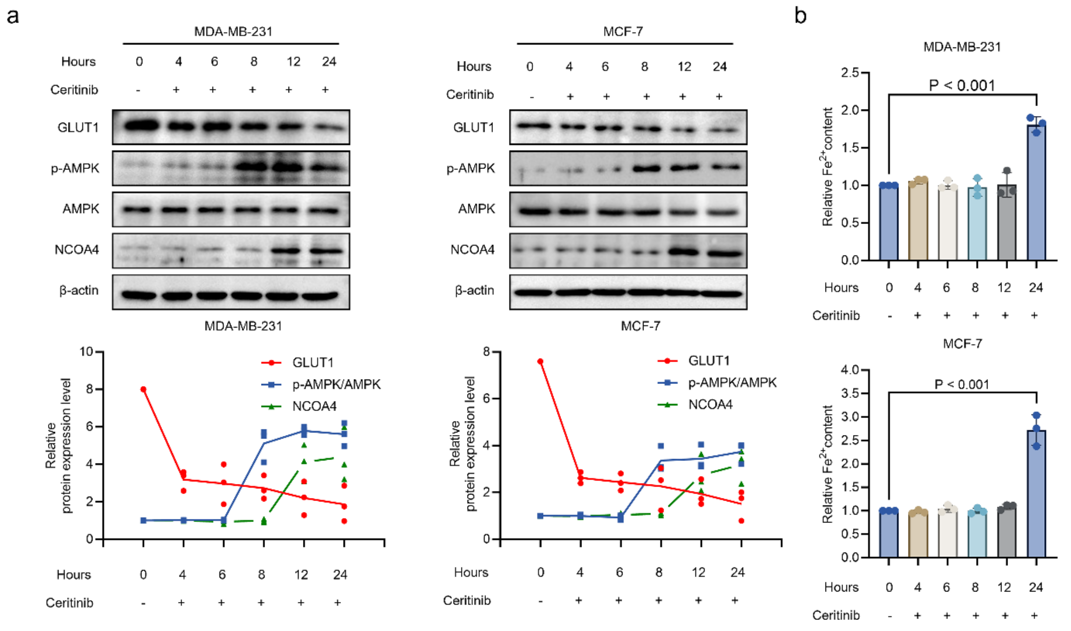

Fig. S6 Ceritinib promotes TRIM21-Mediated GLUT1 degradation and triggers ferroptosis in breast cancer cells

(a) Time-dependent changes in GLUT1, p-AMPK, AMPK, and NCOA4 protein levels in MDA-MB-231 and MCF-7 cells treated with ceritinib over 24 hours (n=3).

(b) Intracellular Fe^{2+} levels were measured at indicated time points over 24 hours (n=3). Statistical significance was assessed by Nonlinear regression, 1-way ANOVA and Tukey's post hoc test. The data are shown as the mean ± SEM.

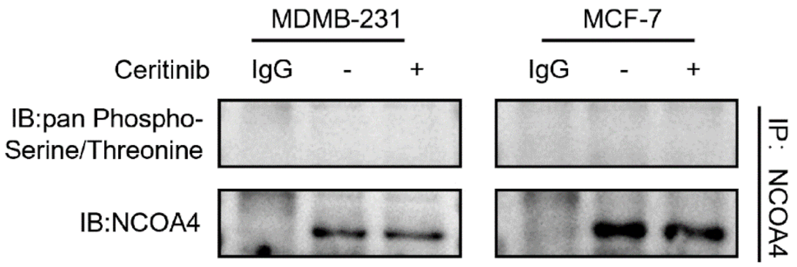

Fig. S7 Ceritinib does not alter the overall phosphorylation status of NCOA4 in breast Cancer Cells

Co-immunoprecipitation (Co-IP) assays were performed in MDA-MB-231 and MCF-7 cells treated with ceritinib or control. Cell lysates were immunoprecipitated with an anti-NCOA4 antibody or IgG control, followed by western bolt with antibodies against NCOA4 and pan-phospho-serine/threonine (n=3).

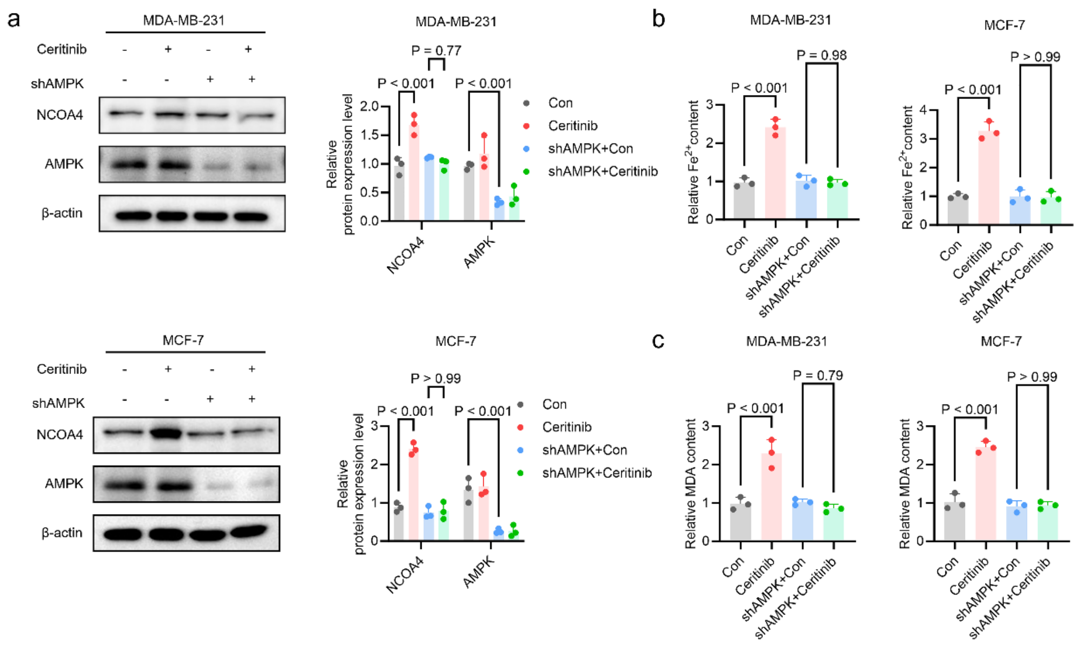

Fig. S8 AMPK is required for ceritinib-induced NCOA4 upregulation and ferroptosis-associated lipid peroxidation

(a) Western blot analysis of NCOA4 and AMPK protein levels in MDA-MB-231 and MCF-7 cells transfected with control shNC or shAMPK and treated with ceritinib or vehicle (n=3).

(b) Relative intracellular Fe^{2+}levels in MDA-MB-231 and MCF-7 cells under the indicated treatments (n=3).

(c) Relative MDA content in MDA-MB-231 and MCF-7 cells under the indicated treatments (n=3).

Statistical significance was assessed by 1-way ANOVA or 2-way ANOVA and Tukey's post hoc test. The data are shown as the mean ± SEM.

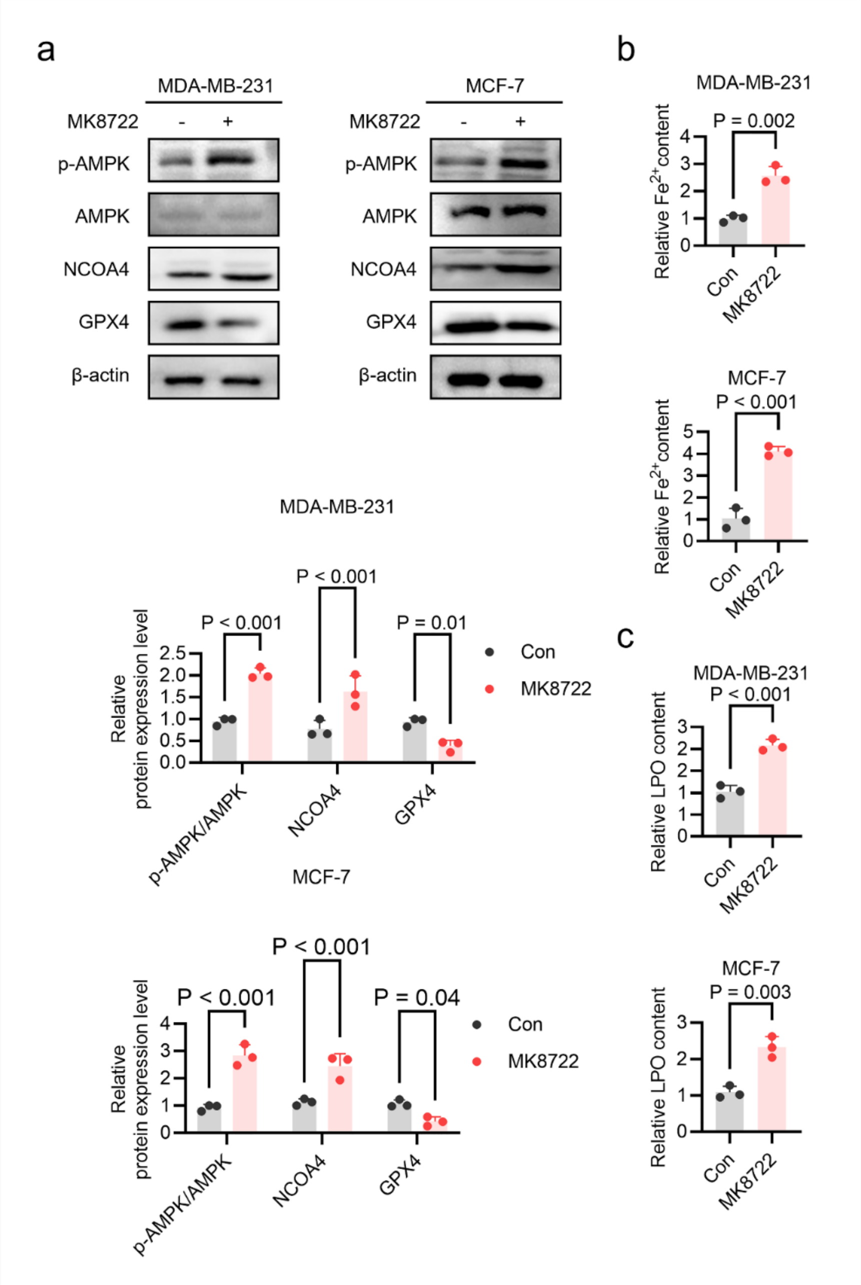

Fig. S9 Pharmacological activation of AMPK is sufficient to trigger ferroptosis-associated molecular events

(a) Western blot analysis of p-AMPK, AMPK, NCOA4 and GPX4 levels in MDA-MB-231 and MCF-7 cells treated with the AMPK activator MK8722 or control (n=3).

(b) Relative intracellular Fe^{2+} levels in MDA-MB-231 and MCF-7 cells following MK8722 treatment (n=3).

(c) LPO levels were measured in breast cancer cells treated with MK8722 (n=3).

Statistical significance was assessed by Student's t-test, 2-way ANOVA and Tukey's post hoc test. The data are shown as the mean ± SEM.

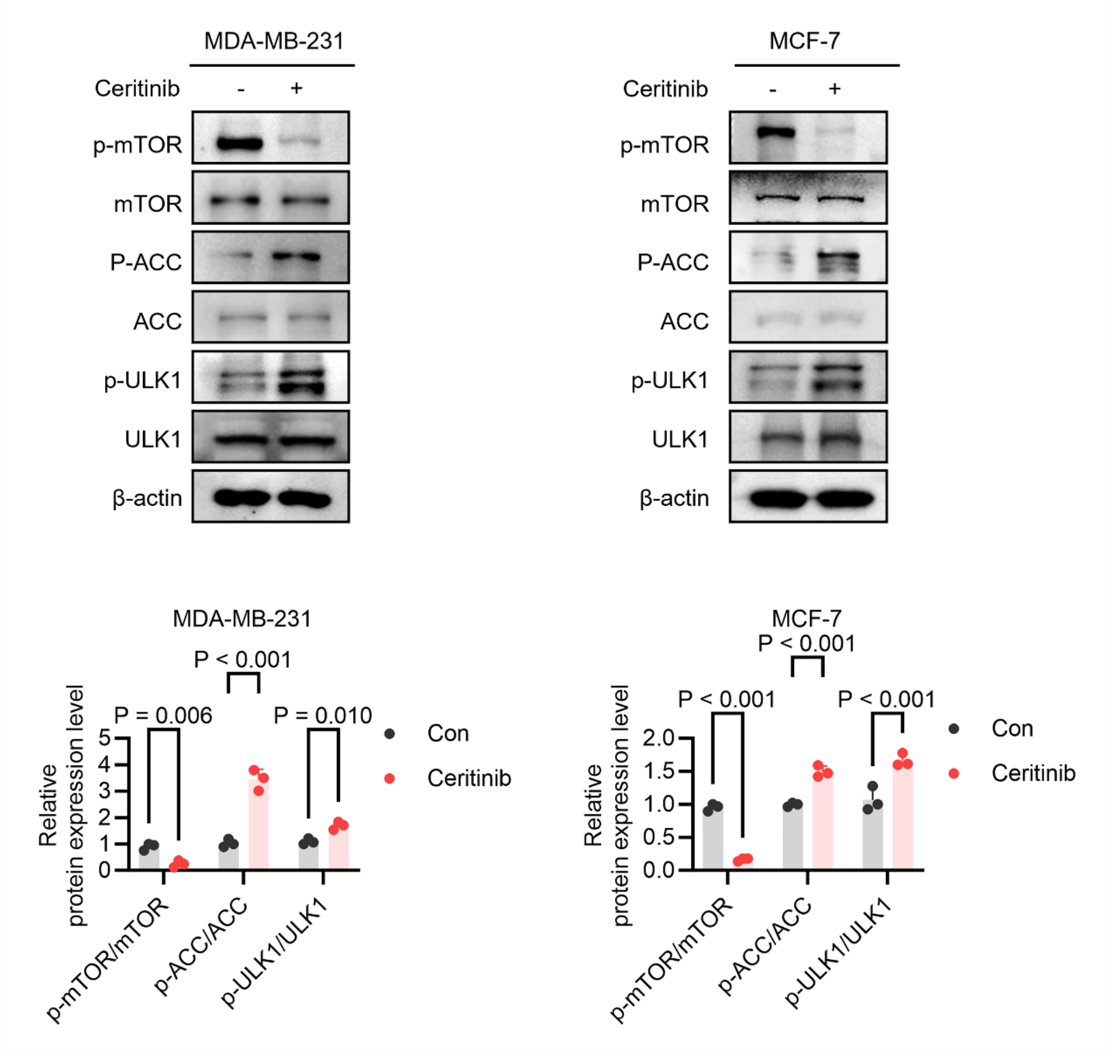

Fig. S10 Ceritinib suppresses mTOR signaling and activates the AMPK-ULK1 axis

Western blot analysis of phosphorylated and total mTOR, ACC, and ULK1 in MDA-MB-231 and MCF-7 cells treated with ceritinib or vehicle control (n=3).

Statistical significance was assessed by 2-way ANOVA and Tukey's post hoc test. The data are shown as the mean ± SEM.

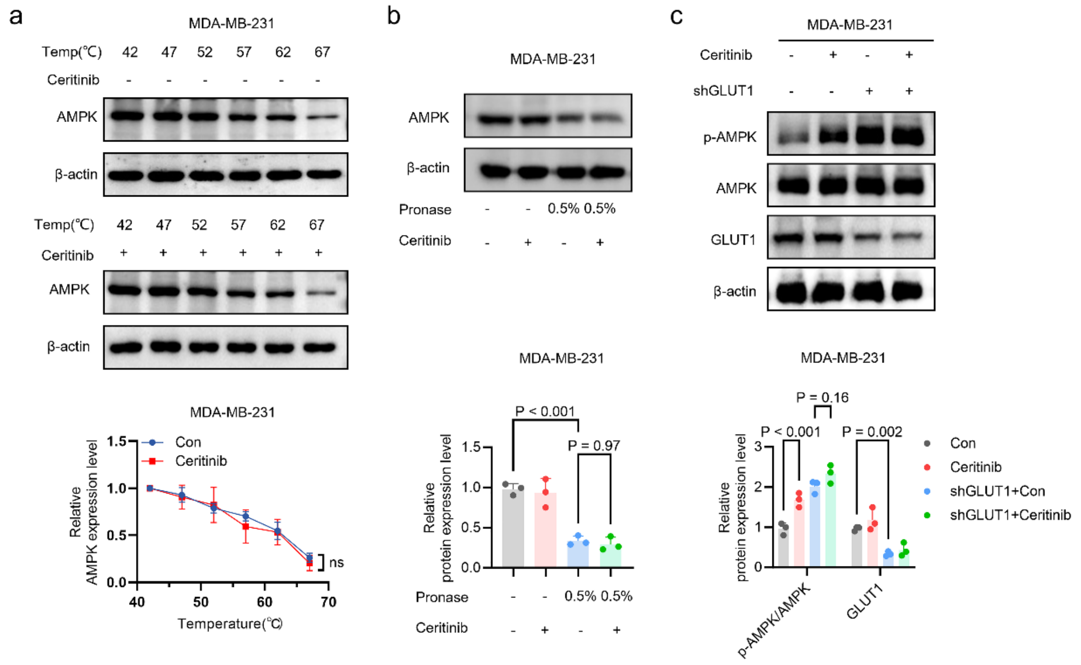

Fig. S11 Ceritinib does not directly bind to AMPK

(a) CETSA was performed in MDA-MB-231 cells treated with ceritinib or control (n=3).

(b) DARTS assay was conducted in MDA-MB-231 cells treated with ceritinib or control (n=3).

(c) Western blot analysis of p-AMPK, AMPK and GLUT1 in MDA-MB-231 cells with or without GLUT1 knockdown (shGLUT1) and ceritinib treatment (n=3).

Statistical significance was assessed by 1-way ANOVA, 2-way ANOVA and Tukey's post hoc test. The data are shown as the mean ± SEM.
